# Changes in the global impact of COVID-19 on nuclear medicine departments during 2020: an international follow-up survey

**DOI:** 10.1007/s00259-021-05444-7

**Published:** 2021-06-19

**Authors:** Francesco Giammarile, Roberto C. Delgado Bolton, Noura El-Haj, Lutz S. Freudenberg, Ken Herrmann, Miriam Mikhail, Olga Morozova, Pilar Orellana, Olivier Pellet, Enrique Estrada L., Sobhan Vinjamuri, Gopinath Gnanasegaran, Yaroslav Pynda, Maria C. Navarro-Marulanda, Partha S. Choudhury, Diana Paez

**Affiliations:** 1 Nuclear Medicine and Diagnostic Imaging Section, Division of Human Health, Department of Nuclear Sciences and Applications, International Atomic Energy Agency, 1400 Vienna, Austria; 2 Department of Diagnostic Imaging (Radiology) and Nuclear Medicine, University Hospital San Pedro and Centre for Biomedical Research of La Rioja (CIBIR), Logroño, La Rioja Spain; 3 ZRN Rheinland and ZRN MVZ GmbH, Korschenbroich, Germany; 4 Department of Nuclear Medicine, University Hospital Essen, Essen, Germany; 5 Nuclear Medicine Department, Liverpool University Hospitals, Liverpool, UK; 6 Nuclear Medicine Department, Royal Free London, London, UK; 7 Nuclear Medicine Department, Rajiv Gandhi Cancer Institute, Delhi, India

**Keywords:** COVID-19, Nuclear medicine, Global impact, Survey

## Abstract

**Aim:**

As a follow-up to the international survey conducted by the International Atomic Energy Agency (IAEA) in April 2020, this survey aims to provide a situational snapshot of the COVID-19 impact on nuclear medicine services worldwide, 1 year later. The survey was designed to determine the impact of the pandemic at two specific time points: June and October 2020, and compare them to the previously collected data.

**Materials and methods:**

A web-based questionnaire, in the same format as the April 2020 survey was disseminated to nuclear medicine facilities worldwide. Survey data was collected using a secure software platform hosted by the IAEA; it was made available for 6 weeks, from November 23 to December 31, 2020.

**Results:**

From 505 replies received from 96 countries, data was extracted from 355 questionnaires (of which 338 were fully completed). The responses came from centres across varying regions of the world and with heterogeneous income distributions. Regional differences and challenges across the world were identified and analysed. Globally, the volume of nuclear medicine procedures decreased by 73.3% in June 2020 and 56.9% in October 2020. Among the nuclear medicine procedures, oncological PET studies showed less of a decline in utilization compared to conventional nuclear medicine, particularly nuclear cardiology. The negative impact was also significantly less pronounced in high-income countries. A trend towards a gradual return to the pre-COVID-19 situation of the supply chains of radioisotopes, generators, and other essential materials was evident.

**Conclusion:**

The year 2020 has a significant decrease in nuclear medicine diagnostic and therapeutic procedures as a result of the pandemic-related challenges. In June, the global decline recorded in the survey was greater than in October when the situation began to show improvement. However, the total number of procedures remained below those recorded in April 2020 and fell to less than half of the volumes normally carried out pre-pandemic.

**Supplementary Information:**

The online version contains supplementary material available at 10.1007/s00259-021-05444-7.

## Introduction

In December 2019, a new type of coronavirus, 2019-nCoV/SARS-CoV-2, the virus that causes coronavirus disease 2019 (COVID-19), was extracted and identified from samples of the lower respiratory tract of patients with atypical pneumonia in Wuhan, China [1–6]. People with COVID-19 exhibited a wide range of symptoms, ranging from mild ones including fever, fatigue, dry cough and headache to serious illness with pneumonia, respiratory distress, multiple organ failure, thrombotic accidents and death. COVID-19 was declared a pandemic by the World Health Organization (WHO) on 11 March 2020 and has since presented the world with unparalleled medical, scientific, social, economic and ethical challenges.

At the time of writing this paper (April 2021), the COVID-19 pandemic is in a phase of transition, with countries in different phases of recovery and some countries experiencing additional peaks. The WHO dashboard indicates over 140 million confirmed cases of COVID-19, including over 3 million deaths [7]. Several countries have begun rolling out targeted vaccination efforts and new COVID variants have been identified [1–3, 7].

This unprecedented health and social crisis have demonstrated that the preparedness of healthcare systems varies greatly in countries around the world. To mitigate the impact of the COVID-19 crisis on essential health services, the WHO published “COVID-19: Operational Guidance for maintaining essential health services during an outbreak” [8] and the International Atomic Energy Agency (IAEA) produced technical guidance to help nuclear medicine departments continue their services and provided health professionals worldwide with related training and resources, including webinars and open-access publications [9–12]. Moreover, in September 2020, the results of a survey on the impact of COVID-19 on nuclear medicine services was published, showing that academic, hospital-based, private, public, inpatient and outpatient facilities, as well as diagnostic and therapeutic services, had been dramatically impacted by the pandemic [13]. Regional differences and challenges in the practice of diagnostic or therapeutic nuclear medicine as well as the availability of medical and financial resources were identified.

In this paper, we present the results of a global web-based survey, conducted by the IAEA and designed to determine the impact of the pandemic on nuclear medicine services worldwide at two specific time points: June and October 2020.

The selection of time points for analysis was based on the stage of the pandemic. Although there were differences in the timing of the COVID-19 waves between regions, most countries were in a post-peak phase at the beginning of the summer (June), while in Autumn (October), most were experiencing the second wave of the pandemic [7]. In 1999, the WHO established a six-phase classification for pandemics, a classification that was maintained in the 2005 revision [14]. In phase 1, there is no evidence that a virus circulating in animals causes infections in humans. In phase 2, a virus circulating in domestic or wild animals is known to have caused infection in humans and is therefore considered a potential pandemic threat. In phase 3, the virus has caused sporadic cases or small clusters of human cases but has not caused enough person-to-person transmission to sustain community outbreaks. In phase 4, there is person-to-person transmission and community outbreaks can occur, indicating a significantly increased risk to become a pandemic. Phase 5 is characterized by the spread of the virus from person-to-person in at least two countries in one WHO region, the possibility of a pandemic is imminent and immediate mitigation efforts are required. In phase 6, the community outbreaks are presented in countries of two WHO regions; the designation of this phase indicates that a global pandemic is occurring. During the post-peak period, disease levels in most countries with adequate surveillance will have fallen below the maximum levels observed. The period after the peak means that pandemic activity appears to be declining; however, it is not known if there will be new waves. In the post-pandemic period, the disease will have returned to normally observed levels; at this stage, it is important to maintain vigilance.

## Materials and methods

As a follow-up of the survey conducted last year spanning April—May 2020 and published in the *Journal of Nuclear Medicine* in September 2020 [13], an invitation to participate in a web-based questionnaire was extended to nuclear medicine facilities worldwide. The survey was made available online for 6 weeks, from November 23 to December 31, 2020.

Efforts were undertaken to ensure extensive and diverse site inclusion worldwide, including both private and public health care organizations, and practices of different sizes. Outreach to encourage participation from sites performing was undertaken through a variety of methods. These included emails from the IAEA to all departments who had participated in the first survey. In addition, potential participants and contacts including those registered in IAEA-compiled databases of health care facilities, NUMDAB [15], and past participants in IAEA research and educational projects (such as technical cooperation projects or regional training courses) received an invitation to participate in the survey. Other modes of distribution included communications shared by professional societies to their members and an outreach campaign through the IAEA’s social media channels including Linkedin, Facebook and Twitter, encouraging dissemination and sharing with nuclear medicine departments.

Based on the IAEA standardized methodology, a web-based data entry system was devised to collect data on the impact around the world of the COVID-19 pandemic on nuclear medicine practices. The IAEA employs a secure software platform, the International Research Integration System, for questionnaire data collection (IRIS, https://iris.iaea.org).

Questionnaires addressed the following categories: operational aspects of nuclear medicine facilities, impact on diagnostic and therapeutic nuclear medicine procedures, availability of personal protective equipment (PPE), and supply of radiotracers and other essential materials. No patient-specific or confidential data were collected, and all participation by study sites was voluntary; therefore, it was deemed that no external ethics committee review was required, and the study complies with the Declaration of Helsinki.

All survey responses were reviewed and checked for missing data, implausible-appearing data and duplicate or inconsistent entries from the same institution. Entries were compiled in Excel for Microsoft 365 (Microsoft, Redmond, Washington). Maps were created using Mapbox. Responses to open-ended questions were collected separately.

Where applicable, we report mean and median results (as well as ranges where necessary). Results presented herein are aligned with the aforementioned categories.

## Results

### General

**Fig. 1:**
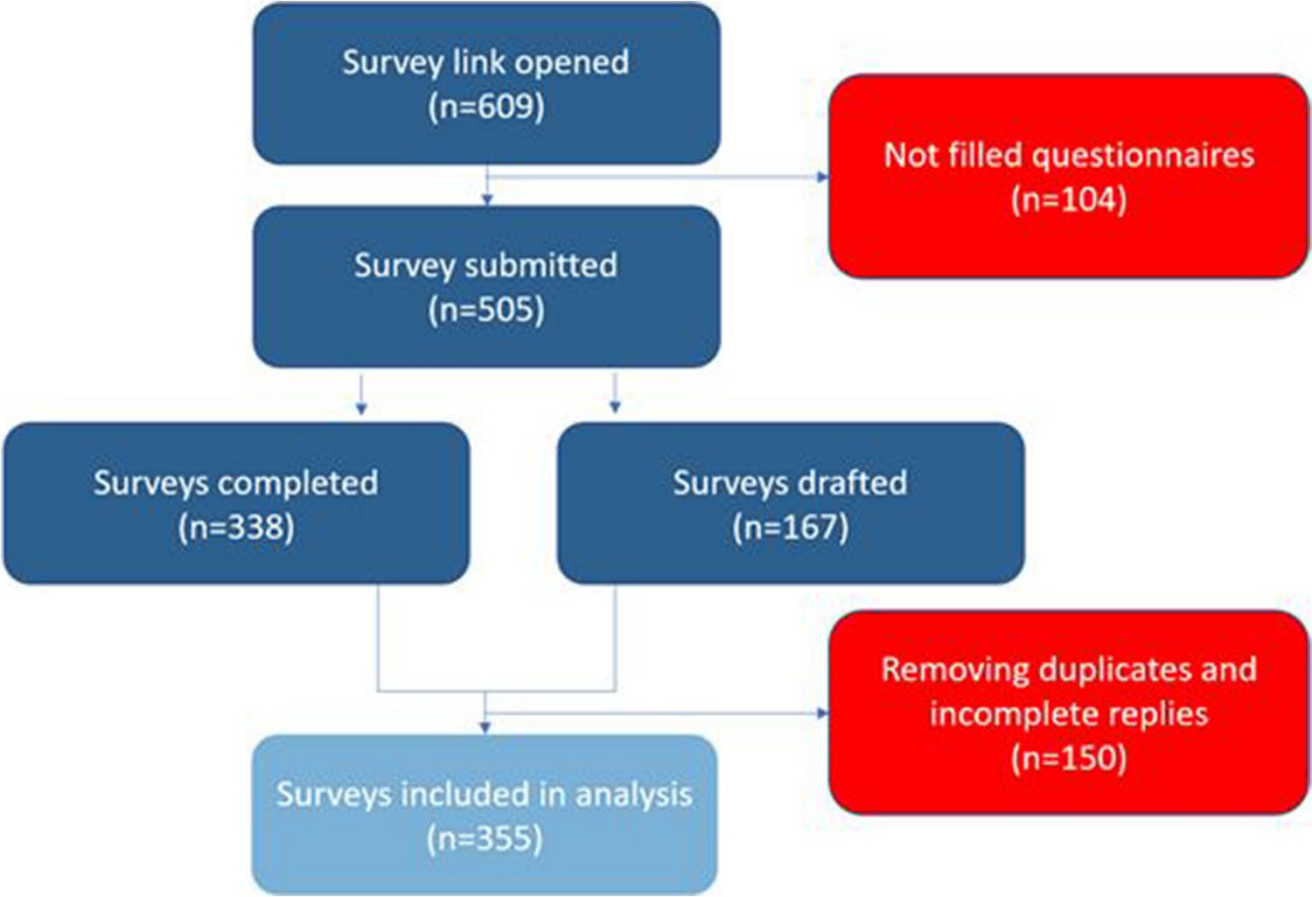
Flow diagram of survey completion

A total of 505 responses were received from 96 countries of which 338 questionnaires were completed in their entirety; of the remaining 167 entries, which were not fully completed, 17 presented useful data. In total, data included in 355 questionnaires were analysed (Fig. 1) (slightly lower than the 434 responses evaluated from 72 countries in the previous survey in 2020).

**Fig. 2:**
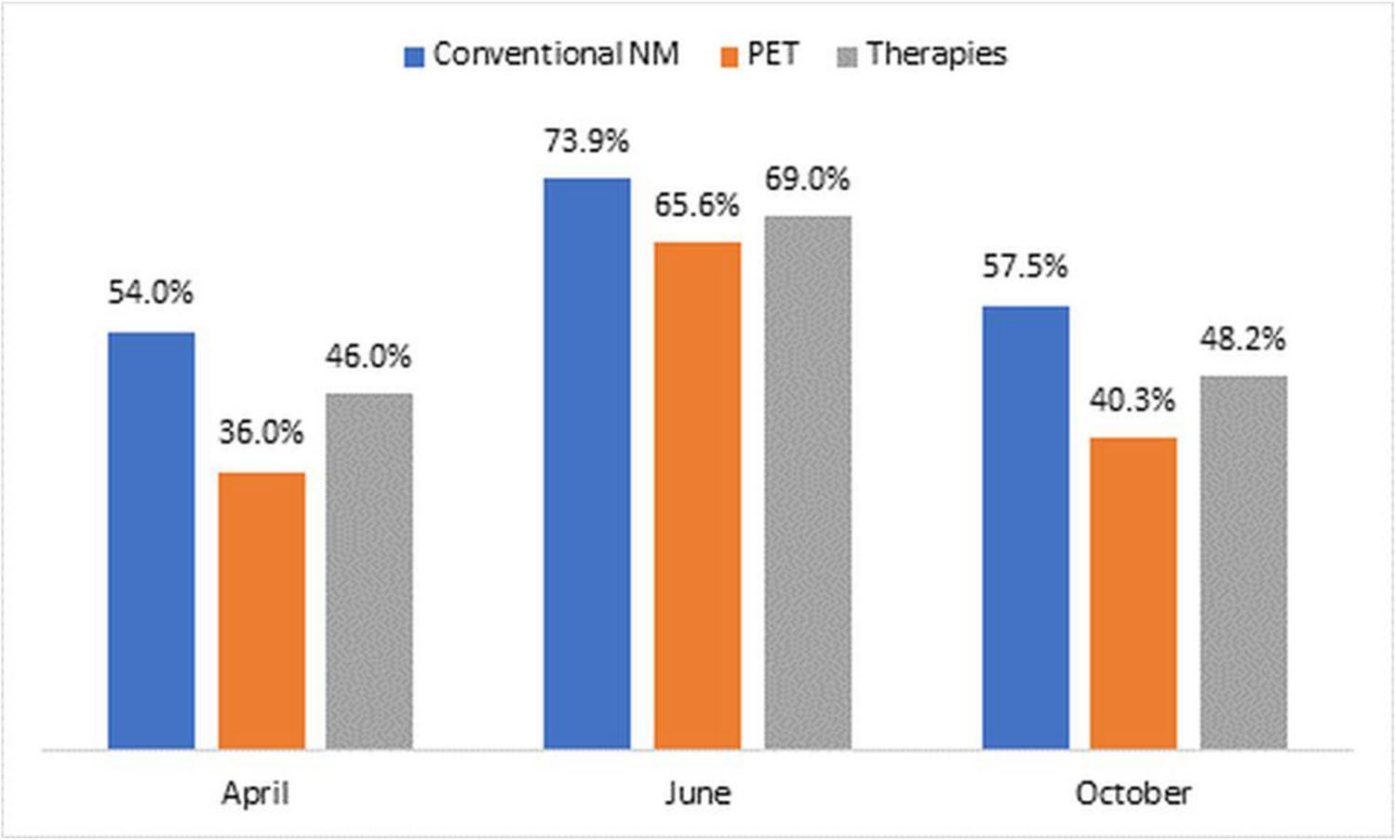
Decrease in nuclear medicine procedures in April [13], June and October 2020

Globally, the volume of nuclear medicine procedures (diagnostic and therapeutic) decreased by 73.3% in June 2020 and 56.9% in October 2020 (the decrease in April 2020 was 54.4% for diagnostic procedures and 45% for therapeutic procedures, as reported in the previous survey), compared to the average number of procedures performed before the COVID-19 pandemic. When analysing by procedure, the reductions affected conventional nuclear medicine by 73.9% and 57.5% in June and October respectively (54% in April 2020), PET by 65.6% and 40.3% (36% in April 2020) and radionuclide therapies by 69.0% and 48.2% (46% in April 2020). Overall, the results of this survey showed a greater decline in all the nuclear medicine procedures when compared to the data reported by the IAEA in a survey conducted in April 2020 [13] (Fig. 2).

### Distribution

For the analysis, data from participating countries were divided into four geographic regions: Africa, Asia and the Pacific, Europe and Latin America and the Caribbean, using the regional definitions of the IAEA’s Technical Cooperation Programme [11] and by income status.

**Fig. 3:**
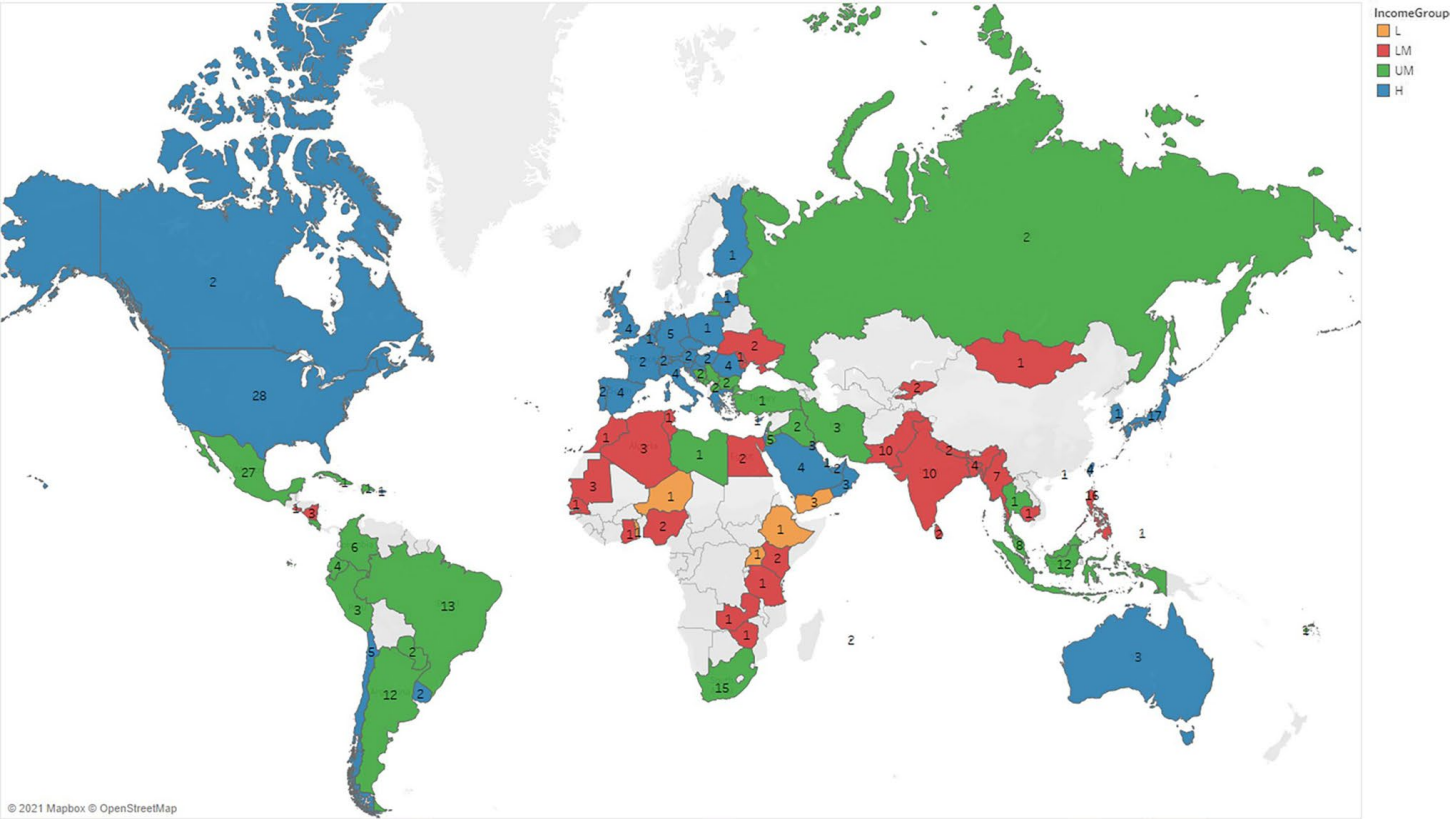
Geographic and economic distribution of the 355 participating centres

Of the 355 institutions included in the analysis (434 in the survey of April 2020), 11.5% (24/434 or 5.5% in the survey of April 2020) came from Africa, 31.8% from the Americas (47/434 or 10.8% in the survey of April 2020), 38.8% from Asia–Pacific (114/434 or 26.3% in the survey of April 2020) and 17.8% from Europe (88/434 or 20.3% in the survey of April 2020) and when divided by income, 2.0% were from low-income countries (LICs), 22.9% from lower-middle-income countries (LMICs), 37.5% from upper-middle-income countries (UMICs) and 37.7% from high-income countries (HICs) (Fig. 3).

Low-income economies are defined as those with a Gross National Income (GNI) per capita, calculated using the World Bank Atlas method, of $1035 or less in 2019; lower-middle-income economies are those with a GNI per capita between $1036 and $4045; upper-middle-income economies are those with a GNI per capita between $4046 and $12,535 and high-income economies are those with a GNI per capita of $12,536 or more [16].

### Respondents

Seventy-one percent of the respondents were nuclear medicine physicians (85% in the survey of April 2020), 9% were nuclear medicine technologists, 7% medical physicists, 5% radiologists (3% in the survey of April 2020) and 8% others (mainly radiopharmacists) (the miscellanea group including medical physicists, technologists, radiopharmacists and others comprised 12% in the survey of April 2020). Forty-four percent of the participants worked in community hospitals (34% in the survey of April 2020), 36% were university-based employees (49% in the survey of April 2020) and 20% worked in private practice (17% in the survey of April 2020).

Only 18% of respondents to this second survey (October 2020) had also participated in the first (April 2020).

### Impact on nuclear medicine diagnostic procedures

#### Share of outpatients

On average, 70% (median, 80%) of all services provided by participating centres were for outpatients (average 74.5%, median 80% in the survey of April 2020). Table 1 shows variability in the proportion of outpatients’ visits in a centre-based analysis, with overall values showing a decrease in 69.9% in June and 49% in October, an increase of 8.5% in June and 17.5% in October, no changes in 17.5% in June and 29% in October and not available in 4.1% in June and 4.5% in October.

**Table 1:** Variability in the proportion of outpatients’ visits in a centre-based analysis per region in percentages of the total 355 questionnaires

Regions	June (%)				October (%)			
	-	+	=	?	-	+	=	?
Africa	8.9	0.3	1.1	1.1	5.0	3.1	2.3	1.1
North America	5.6	0.3	2.3	0.3	3.9	1.9	2.3	0.3
Latin America and the Caribbean	17.7	2.2	2.8	0.5	13.2	4.2	5.3	0.5
Far East	7.0	0.9	2.8	0.9	5.3	1.1	4.2	0.9
Middle East and South Asia	13.2	1.4	1.7	0.5	8.7	3.1	4.2	0.9
South East Asia and the Pacific	7.9	0.9	1.7	0.0	5.9	1.1	3.4	0.0
Eastern Europe	5.0	1.1	1.4	0.3	4.2	1.1	2.3	0.3
Western Europe	4.2	1.4	3.7	0.5	2.5	1.8	5.0	0.5
Total	69.9	8.5	17.5	4.1	49.0	17.5	29.0	4.5

-: decrease

+ : increased

= : stable

?: don’t know

When analysing the variations in the proportion of outpatients’ visits based on a regional analysis, the higher reductions were reported in Latin America and the Caribbean (17.7% in June and 13.2% in October) and the Middle East and South Asia (13.2% in June and 8.7% in October), whereas the lowest reductions were reported in Western Europe (4.2% in June and 2.5% in October), Eastern Europe (5.0% in June and 4.2% in October) and North America (5.6% in June and 3.9% in October).

Regarding the analysis of variations in the proportion of outpatients’ visits based on the income groups, the higher reductions were reported in upper-middle-income countries (29.6% in June and 20.3% in October) and high-income countries (22.6% in June and 14.9% in October), whereas the lowest reductions were reported in low-income countries (1.4% in June and 1.1% in October) and lower-middle-income countries (16.3% in June and 12.7% in October).

#### Conventional nuclear medicine procedures

Of the responding centres, 301 perform conventional nuclear medicine diagnostic procedures of which 1% occurs in LICs, 23% in LMICs, 36.5% in UMICs and 39.5% in HICs.

**Fig. 4:**
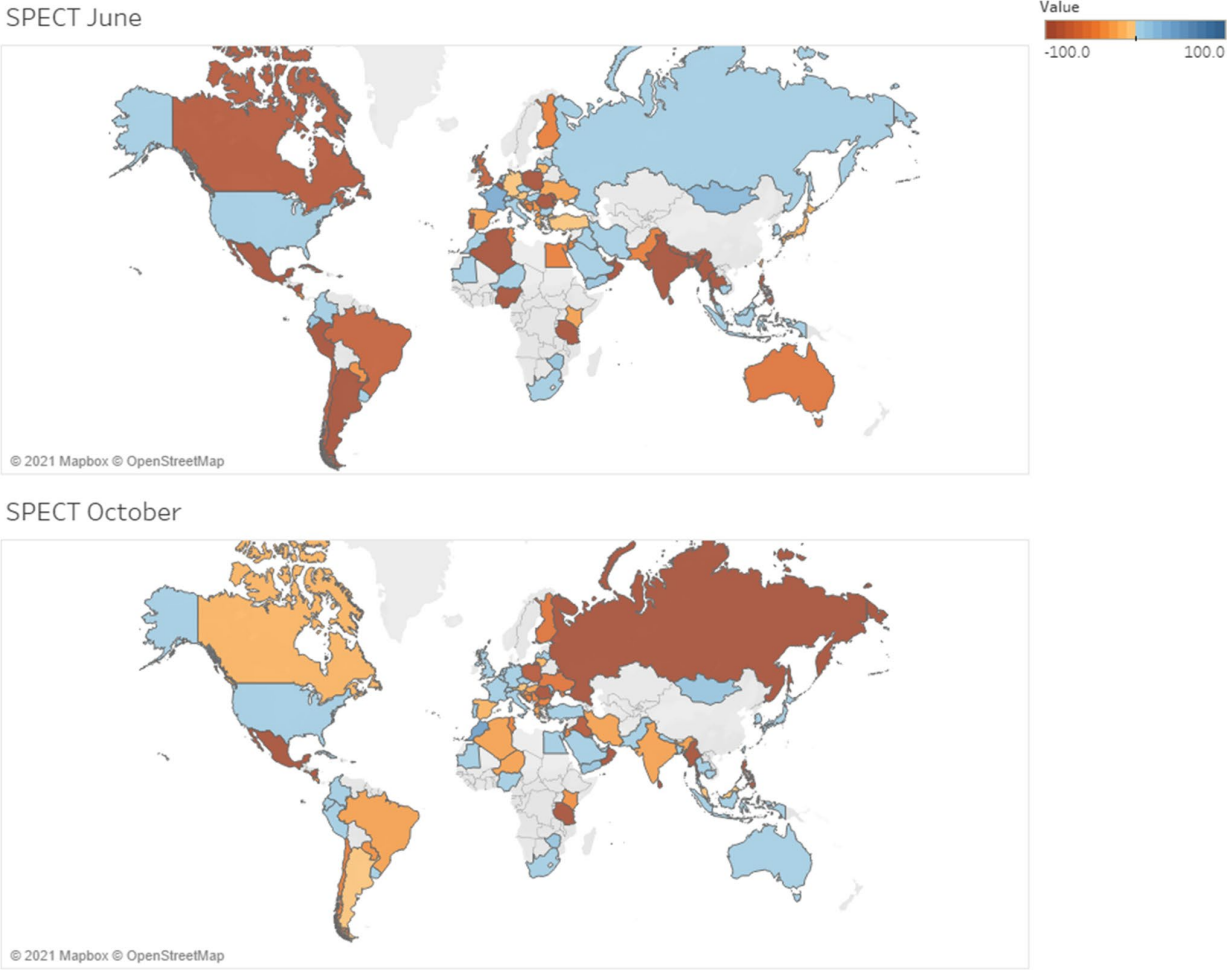
Average variation in conventional nuclear medicine diagnostic procedures by country in June and October 2020

Compared to a pre-COVID-19 baseline, the utilization of conventional nuclear medicine diagnostic procedures changed dramatically in June and October 2020. Globally, decreases were more evident in June (73.9%) rather than October (57.5%) 2020 (54% decrease in the survey of April 2020). The greatest impact was seen in the Americas in June and in the Eastern European countries in October, while Western European countries were less affected. Similarly, the impact was more evident in LMICs than in HICs (Table 2). Regarding the type of studies, the major decreases were observed in myocardial perfusion imaging in June and in bone scans in October (Fig. 4).

**Table 2:** Variation per income groups in percentage of the 355 total answers

Income groups	June (%)				October (%)			
	-	+	=	?	-	+	=	?
Low income	1.4	0.3	0.0	0.3	1.1	0.5	0.0	0.3
Lower middle income	16.3	2.0	2.5	2.0	12.7	3.7	4.8	1.7
Upper middle income	29.6	3.4	3.7	0.7	20.3	6.7	9.6	0.8
High income	22.6	2.8	11.3	1.1	14.9	6.6	14.6	1.7
Total	69.9	8.5	17.5	4.1	49.0	17.5	29.0	4.5

-: decrease

+ : increase

= : no variation

?: not available

#### PET/CT procedures

The responding centres performing PET procedures were 182, distributed 0% in LICs, 16% in LMICs, 40% in UMICs and 44% in HICs.

**Fig. 5:**
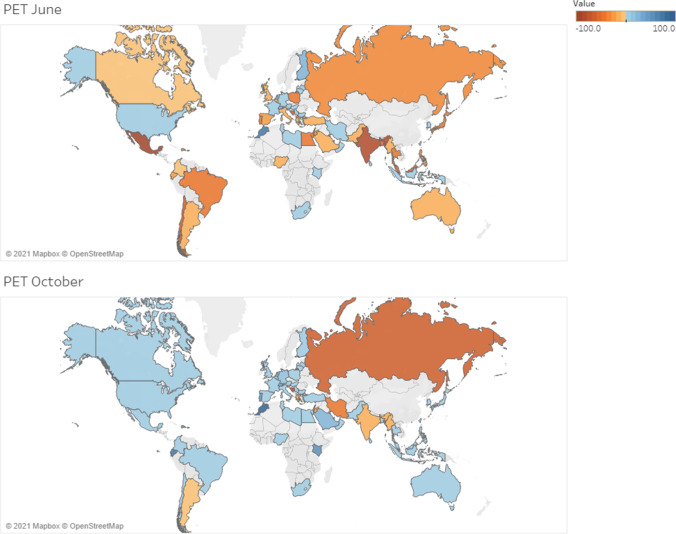
Average variation in PET diagnostic procedures by country in June and October 2020

Compared to activity pre-COVID-19, PET/CT procedures decreased in June (65.6%) and October 2020 (40.3%) (36% decrease in the survey of April 2020). Decreases affected mainly Latin America and South East Asian countries in June for all PET procedures. In October, the situation showed global recovery. As with conventional nuclear medicine activity, the impact was more evident in LMICs both in June and in October, with persistent differences during the recovery phase. For oncological PET studies, in June, the decrease in HICs was 53.5% versus 15% in October. In LMICs, the decrease was 89% in June and 46.7% in October. Non-oncologic studies were affected slightly more than oncologic studies. Gallium activity dramatically decreased in Latin America (93.8% in June) and Asia, while North America and Western Europe were less affected (Fig. 5).

### Impact on radionuclide therapies

The responding centres performing radionuclide therapies were 301, distributed 1% in LICs, 23% in LMICs, 36.5% in UMICs and 39.5% in HICs.

**Fig. 6:**
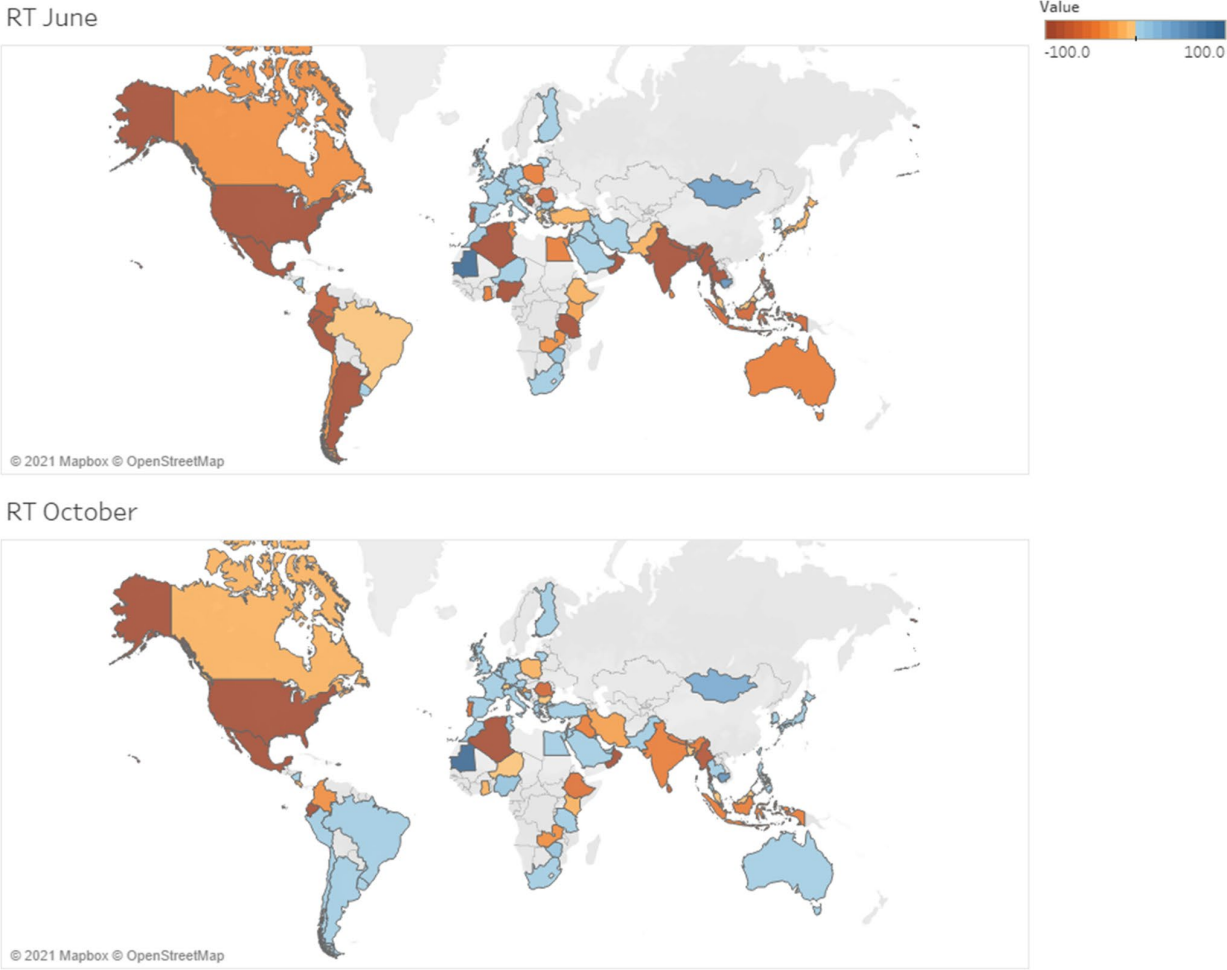
Average variation in therapeutic procedures by country in June and October 2020

Compared to activity pre-COVID-19, radionuclide therapies changed dramatically in June (69.0%) and October (48.2%) 2020 (46% decrease in the survey of April 2020). Decreases affected LMICs more significantly than other regions, mainly in June (Fig. 6).

### Personal protective equipment (PPE)

Thirty-five percent of the respondents reported a shortage of PPE in June 2020. This percentage dropped to 25% in October 2020 (50% decrease in the survey of April 2020). As for the availability of PPE, 32% of sites reported that stockpiles of PPE would last for more than 1 month while fewer than 2 days for only 7% (83% of sites reported that stockpiles of PPE would last for only one month in the survey of April 2020).

**Fig. 7:**
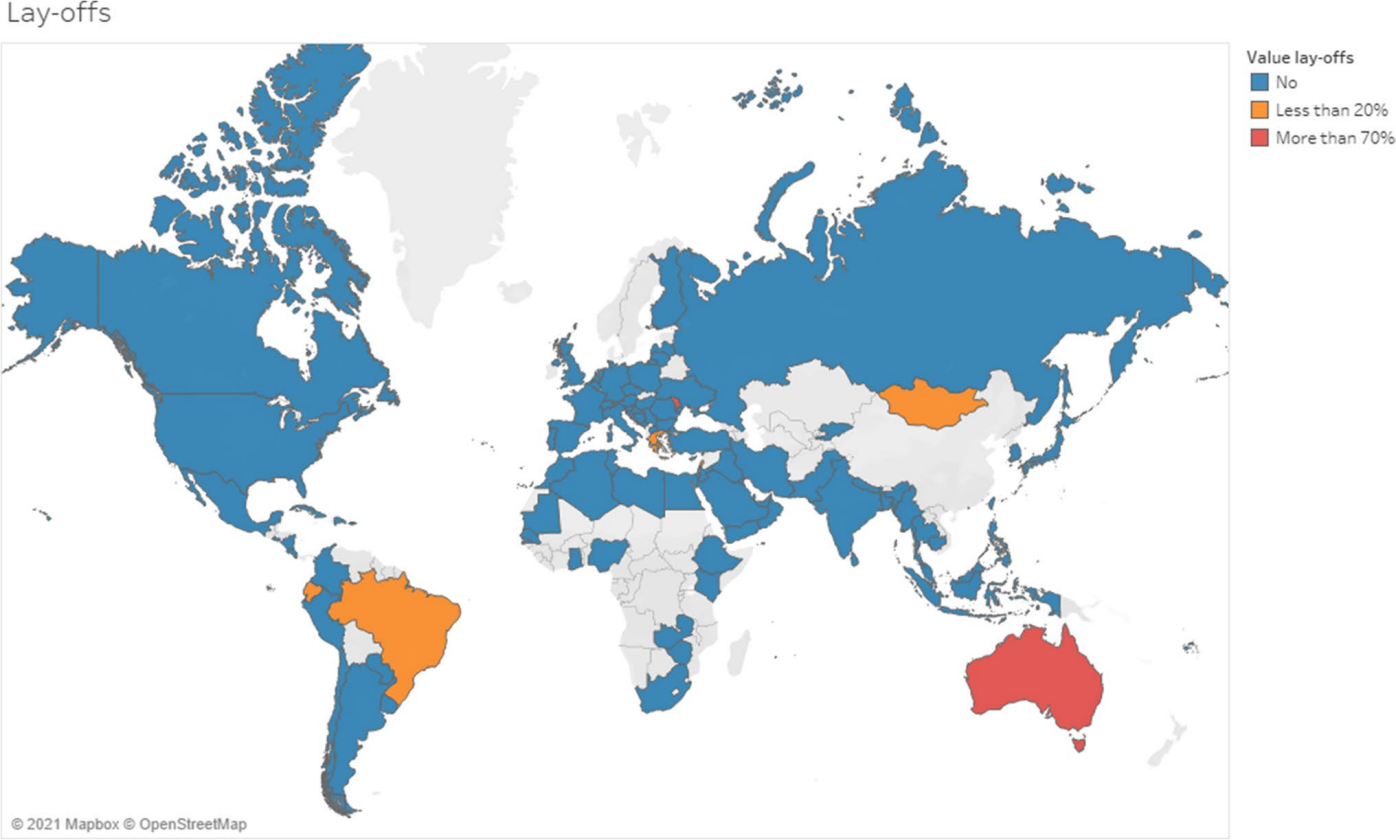
Average variation in the shortage of PPE by country in June and October 2020

Figure 7 shows variability in the shortage of PPE. Decreases affected mainly the Latin American region and LMICs.

### Employee health and organizational changes

Sixty percent (15% in the survey of April 2020) of respondents experienced COVID-19 infections within their own departments: 40% (12% in the survey of April 2020) reported that less than 20% of staff were infected, whereas 14% (2.5% in the survey of April 2020) reported infection rates between 20 and 40%, and 6% (0.5% in the survey of April 2020) observed rates exceeding 40%.

Thirty-one percent of the respondents experienced a relocation of their employees (34% in the survey of April 2020), and in 3% of the cases, the number of relocated employees exceeded 40%. However, in most cases, the relocated employees returned to the nuclear medicine service in October 2020.

**Fig. 8:**
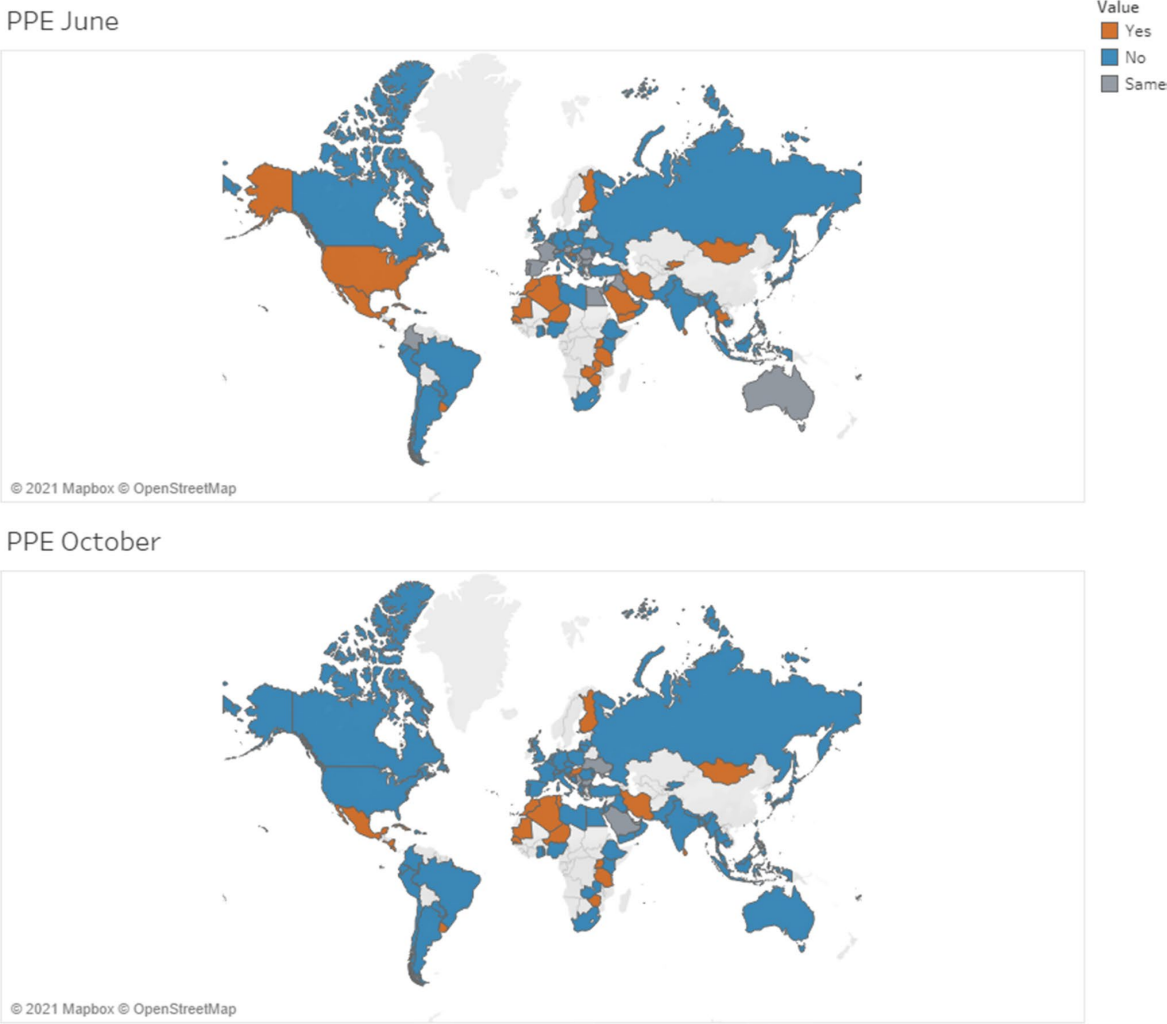
Employment status

More than half of respondents modified working hours (15% in the survey of April 2020); in 16% of the cases for less than 20% of their staff (short, part-time or turnover); 22% (26% in the survey of April 2020) modified their work schedule between 20 and 70%, and 14% (18% in the survey of April 2020) modified working hours by more than 70%. In approximately 20% of the cases, the employees were laid off (Fig. 8).

### Demand and supplies of materials

#### Demand

Approximately half of the respondents modified orders of ^99m^Tc generators (50% reduced orders in the survey of April 2020); of these, 11% (12% in the survey of April 2020) maintained their orders for more than 70% of their regular demand, 22% (25% in the survey of April 2020) maintained between 20 and 70% of their orders and 8% (13% in the survey of April 2020) maintained less than 20% of their orders. Another 7% (12% in the survey of April 2020) cancelled their generator orders entirely.

#### Supply

Insufficient supplies of radioisotopes, generators and kits were reported especially for ^131^I and ^99m^Tc generators. The reduction of essential supplies varied significantly between regions and was more frequently reported from Africa, Asia, Oceania and Latin America (Fig. 9).

## Discussion

The coronavirus disease 2019 (COVID-19) pandemic has presented health care systems worldwide with significant challenges. The majority of countries have experienced a lockdown and a shift in the allocation of healthcare resources to cope with the increased burden of COVID-19 cases. In many regions, the healthcare systems have been stressed to their limits, having to reduce non-urgent procedures and concentrate all available resources to attend to COVID-19 patients. In doing so, the global provision of in- and outpatient services for other diseases have been compromised to varying degrees, including by the necessity to implement additional infection prevention and control measures [17–19].

The impact of the pandemic on nuclear medicine departments and their services worldwide was recently assessed through a survey conducted by the IAEA [13]. It showed a global decrease in procedures performed in April 2020; studies done with ^99m^Tc decreased by 54%; PET studies decreased by 36% and radionuclide therapies by 45% on average. The main reasons for this decline were attributed to the postponement of non-emergent procedures and to the disruption in the supply chain, mainly affecting LMICs.

Today, the pandemic and the associated lockdown have redesigned the usual risk/benefit ratio, which compares the infection exposure risk (for the patient and for staff) vs. the risk associated with delaying or cancelling nuclear medicine procedures. As we enter another phase of the pandemic, plans have been initiated to restore the provision of deferred services.

According to the IAEA medical imaging and nuclear medicine global resources database—IMAGINE [20], there are approximately 27,000 SPECT scanners available worldwide, of which, less than 0.1% are installed in LICs, 3% in LMICs, 16.5% in UMICs and 80.3% in HICs. Regarding PET scanners, there are approximately 5700 scanners available, of which less than 0.1% are installed in LICs, around 8% in LMICs, 15% in UMICs and 77% in HICs [20].

The aim of this new IAEA online survey was to measure the impact of COVID-19 on nuclear medicine departments at two fixed times in 2020, namely June and October. The selection of temporal baselines for analysis was based on the stage of the pandemic. In June, most countries were in a post-peak phase, while in October most were experiencing a new wave.

It was noted that the decline of nuclear medicine procedures in June was greater than in October and higher than what was reported in the survey conducted in April 2020 [13], at the beginning of the pandemic, and still far from returning to average numbers of procedures before COVID-19.

Although the results of this follow-up survey cannot be directly compared with the previous one [13], less than 20% of the responses were repeated respondents from the first survey. Nevertheless, the larger geographic and income distributions allowed an overview that is more representative of the global impact of COVID-19 on the practice of nuclear medicine.

The reason for this lower response rate could be attributed to the lower motivation of potential respondents to repeat the survey and to the great number of surveys and virtual events that have been deployed during the pandemic, causing virtual fatigue. Thus, only June and October 2020 could be compared herein. Recovery phases are not discussed as regions differed significantly in this regard.

A total of 505 centres from 96 countries responded to the questionnaire. However, after the removal of duplicates and insufficient replies, available data could be extracted from 355 questionnaires. The received responses came from institutions uniformly distributed across the world and according to income status. This highly balanced distribution among participating centres was not obtained in our previous survey, where the responses from high-income countries were preponderant [13].

Globally, the volume of nuclear medicine procedures (diagnostic and therapeutic) decreased by 73.3% in June 2020 and 56.9% in October 2020, compared to the average number of procedures performed before the COVID-19 pandemic, varying markedly between regions and countries, with the greatest regional decreases in Latin America. Significant reductions affected about 80% and 60% of conventional diagnostic procedures, 65% and 40% of PET and 70% and 50% of therapeutic procedures in June and October, respectively. The biggest impact was seen in low- to low-middle-income countries, both in June and October (despite the partial recovery), while high-income countries exhibited greater continuity of nuclear medicine services since the outset of the pandemic. This observation was also seen in a recent national survey examining the impact of COVID-19 on nuclear medicine services in 2020 compared to 2019 [21]. The initial significant reduction in nuclear medicine procedures in the first 3 weeks of the COVID-19 pandemic did not continue, but there was no recovery of the previously discontinued services [22]. The decrease in diagnostic and therapeutic procedures of benign diseases was particularly severe [21].

Similar to the interpretation of the April 2020 survey, this decrease could be attributed to several factors such as changes in scheduling workflow with a reduction in the number of appointments, reluctancy of patients to visit a medical centre due to safety concerns, deferral of non-urgent studies, deferral of surgeries and pre- or perioperative evaluations, shortages of essential supplies, implementation of infection prevention and control (IPC) measures including social distancing and decreasing the number of health workers at a given time to reduce staff exposure, and increasing the time assigned to each patient to include greater disinfection and cleaning procedures [13].

Also, the decline in diagnostic tests was more pronounced for conventional nuclear medicine studies (thyroid, cardiac, bone and lung scans) than for PET/CT scans. This may be for two reasons: first, PET tracers are produced through local cyclotrons, whereas most of the countries rely on international flights for the supply of ^99m^Tc/^99^Mo generators and other radioisotopes and, second, the more urgent nature of cancer assessments is with PET/CT.

Regarding clinical indications for referral to both conventional nuclear medicine and PET/CT, the greatest reduction in exams was seen for myocardial perfusion and non-oncological studies. This could be attributed to the trepidation of patients fearing exposure to the virus in a healthcare setting and, as above, to certain exams being deemed less urgent, a pragmatic choice in this period of crisis. Compared to the previous survey [13], the global decrease was more pronounced, probably because of the minor impact felt by high-income countries, which represented a larger constituent group in the first survey. Specifically, the changes in the availability of technetium or gallium generators and of radiopharmaceutical delivery in general were less detrimental to the continuity of services in high-income countries whereas low- to low-middle income countries experienced greater insufficiencies in supplies of radioisotopes, generators, and kits.

Notably, some centres reported an increase in their activities, namely 6.5% and 13% for conventional nuclear medicine diagnostic procedures, 10.5% and 21% for PET and 5.5% and 13% for therapeutic procedures in June and October respectively. This aspect, uniformly distributed across the world and according to income status, reflects a trend towards the recovery of previous activity.

As for radionuclide therapies, the main reduction was reported in radioiodine therapies for benign thyroid disease followed by thyroid cancer and radiosynovectomy in June, similar to what was reported in the previous survey [13], and in radiosynovectomy in October. Compared to less affected procedures, such as selective internal radiation therapy, peptide receptor radionuclide therapy and prostate-specific membrane antigen radioligand therapy, the latter are treatments that could be deferred with less impact on patient prognosis.

Adoption of IPC measures is essential to protect health workers and patients while continuing to provide medical services [10, 11, 23–25]. Thus, the availability of PPE is critical. In our survey, 35% and 25% of participants, respectively in June and October 2020, reported a shortage of PPE, with no significant differences among geographic regions. Regarding the 50% rate reported in the previous survey conducted in April [13], the trend of a general return to normality is evident, due to the increase in supplies. Conversely, COVID-19 infections in staff were reported in 60% of surveyed centres, with a great increase in nosocomial infections compared to 15%, reported in April [13].

The overall significant decrease in nuclear medicine procedures also resulted in a reduction of working hours in almost half of the responding centres, affecting large numbers of staff.

The reduction of essential supplies was more frequently reported for Africa, Asia, Oceania, and Latin America. Surveys conducted by international bodies such as the IAEA highlight the need to ensure a uniform and consistent supply of radioisotopes, generators and kits across the globe and to ensure regional imbalances in service delivery are minimized. This can enable the return to pre-pandemic activity levels and hopefully lay the foundation for greater health system strengthening to meet enhanced demands for patient care, as well as for vital educational, research and developmental activity.

## Conclusion

Following the situational snapshot of the COVID-19 impact on nuclear medicine services conducted in April 2020 by the same team of investigators, this second survey in October 2020 aimed to provide a follow-up. The current survey, with 505 responding centres, was more uniformly distributed across the world regions and by country income status, constituting a small but more reliable sampling of existing nuclear medicine centres worldwide.

Our data provides a global perspective of the impact of COVID-19 on nuclear medicine services. Among the different nuclear medicine procedures, PET/CT for oncologic indications showed a lesser decrease in utilization rates than did conventional nuclear medicine and particularly nuclear cardiology. As for radionuclide therapies, the main reduction was reported in radioiodine therapies for benign thyroid disease followed by thyroid cancer and radiosynovectomy. High-income countries exhibited greater continuity of nuclear medicine services since the outset of the pandemic compared to lower-resource settings. The survey also showed a trend in the restitution towards normalcy of the supply chains of radioisotopes, generators and other essential materials.

In June, the global decline recorded in the survey was greater than in October when the situation began to show improvement. However, the total number of procedures remained below those recorded in April 2020 and fell to less than half of the volumes normally carried out pre-pandemic.

## Supplementary Information

Below is the link to the electronic supplementary material.

Supplementary file1 (DOCX 292 KB)

### Supplementary Information

Below is the link to the electronic supplementary material.

Supplementary file2 (DOCX 40.0 KB)

### Supplementary Information

Below is the link to the electronic supplementary material.

Supplementary file3 (PDF 428 KB)

## Abbreviations

COVID-19: Coronavirus disease 2019
CNM: Conventional nuclear medicine
HIC: High-income countries
IAEA: International Atomic Energy Agency
IPC: Infection prevention and control
IRIS: International Research Integration System
LIC: Low-income countries
LMIC: Lower-middle-income countries
PET: Positron emission tomography
PPE: Personal protective equipment
UMIC: Upper-middle-income countries
WHO: World Health Organisation
