# Supplementary material for: Changes in the global impact of COVID-19 on nuclear medicine departments during 2020: an international follow-up survey
Source: Eur J Nucl Med Mol Imaging. 2021 Jun 19;48(13):4318–30. doi: 10.1007/s00259-021-05444-7 (PMC8214460; doi:10.1007/s00259-021-05444-7)

**Annex2: Statistics**

1. **Overall results**

1.1. Distribution of participating institutions by regions

| **Continents** | **Regions** | **Answers** | |
| --- | --- | --- | --- |
|  |  | **#** | **%** |
| Africa | Africa | 41 | 11.5 |
| America | North America | 30 | 8.5 |
|  | Latin America and the Caribbean | 83 | 23.4 |
| Asia and the Pacific | Far East | 41 | 11.5 |
|  | Middle East and South Asia | 60 | 16.9 |
|  | South East Asia and the Pacific | 37 | 10.4 |
| Europe | Eastern Europe | 28 | 7.9 |
|  | Western Europe | 35 | 9.9 |

1.2. Distribution per income group of the 355 participants.

| **Income groups** | **Answers** | |
| --- | --- | --- |
|  | **#** | **%** |
| Low-income | 7 | 2.0 |
| Lower-middle-income | 81 | 22.8 |
| Upper-middle-income | 133 | 37.5 |
| High-income | 134 | 37.7 |

1.3. Variation per regions in percentage of the 355 total responses

Y: shortage
N: no shortage
?: not applicable

| **Regions** | **June (%)** | | | **October (%)** | | |
| --- | --- | --- | --- | --- | --- | --- |
|  | **Y** | **N** | **?** | **Y** | **N** | **?** |
| Africa | 3.4 | 7.0 | 1.1 | 3.1 | 7.3 | 1.1 |
| North America | 4.8 | 3.4 | 0.3 | 2.3 | 5.9 | 0.3 |
| Latin America and the Caribbean | 10.4 | 12.1 | 0.8 | 9.6 | 13.2 | 0.6 |
| Far East | 3.7 | 6.8 | 1.1 | 1.7 | 8.7 | 1.1 |
| Middle East and South Asia | 5.9 | 10.4 | 0.6 | 4.8 | 11.5 | 0.6 |
| South East Asia and the Pacific | 2.8 | 7.0 | 0.6 | 1.1 | 8.7 | 0.6 |
| Eastern Europe | 2.3 | 5.0 | 0.6 | 1.7 | 5.6 | 0.6 |
| Western Europe | 2.0 | 7.9 | 0.0 | 0.8 | 9.0 | 0.0 |
| **Total** | **35.3** | **59.6** | **5.1** | **25.1** | **69.9** | **4.9** |

1.4. Variation per regions in percentage of the respective group responses

| **Regions** | **June (%)** | | | **October (%)** | | |
| --- | --- | --- | --- | --- | --- | --- |
|  | **Y** | **N** | **?** | **Y** | **N** | **?** |
| Africa | 29 | 61 | 10 | 27 | 63 | 10 |
| North America | 57 | 40 | 3 | 27 | 70 | 3 |
| Latin America and the Caribbean | 44 | 52 | 4 | 41 | 57 | 2 |
| Far East | 32 | 58 | 10 | 15 | 75 | 10 |
| Middle East and South Asia | 35 | 62 | 3 | 28 | 69 | 3 |
| South East Asia and the Pacific | 27 | 68 | 5 | 11 | 84 | 5 |
| Eastern Europe | 29 | 64 | 7 | 21 | 72 | 7 |
| Western Europe | 20 | 80 | 0 | 9 | 91 | 0 |

1.5. Variation per income groups in percentage of the 355 total responses

| **Income groups** | **June (%)** | | | **October (%)** | | |
| --- | --- | --- | --- | --- | --- | --- |
|  | **Y** | **N** | **?** | **Y** | **N** | **?** |
| Low-income countries | 1.1 | 0.8 | 0.0 | 0.8 | 1.1 | 0.0 |
| Lower-middle-income countries | 8.2 | 13.0 | 1.7 | 5.9 | 15.2 | 1.7 |
| Upper-middle-income countries | 13.0 | 22.2 | 2.2 | 11.3 | 24.2 | 2.0 |
| High-income countries | 13.0 | 23.7 | 1.1 | 7.0 | 29.6 | 1.1 |
| **Total** | **35.3** | **59.7** | **5.0** | **25.0** | **70.1** | **4.8** |

1.6. Variation per income groups in percentage of the respective group responses

| **Income groups** | **June (%)** | | | **October (%)** | | |
| --- | --- | --- | --- | --- | --- | --- |
|  | **Y** | **N** | **?** | **Y** | **N** | **?** |
| Low-income countries | 57 | 43 | 0 | 43 | 57 | 0 |
| Lower-middle-income countries | 36 | 57 | 7 | 26 | 67 | 7 |
| Upper-middle-income countries | 35 | 60 | 5 | 30 | 65 | 5 |
| High-income countries | 34 | 63 | 3 | 19 | 78 | 3 |

1. **Conventional Nuclear Medicine**

2.1. Worldwide availability of SPECT scanners per income status

| Income groups | Number of SPECT scanners | SPECT scanners / million inhabitants | \| **% of total SPECT scanners** \| \| --- \| \|  \| |
| --- | --- | --- | --- | --- | --- |
| Low-income | 21 | 0.04 | 0.08 |
| Lower-middle-income | 818 | 0.281 | 3.01 |
| Upper-middle-income | 4,492 | 1.574 | 16.54 |
| High-income | 21,825 | 17.645 | 80.37 |

2.2. Average variation in conventional nuclear medicine diagnostic procedures globally
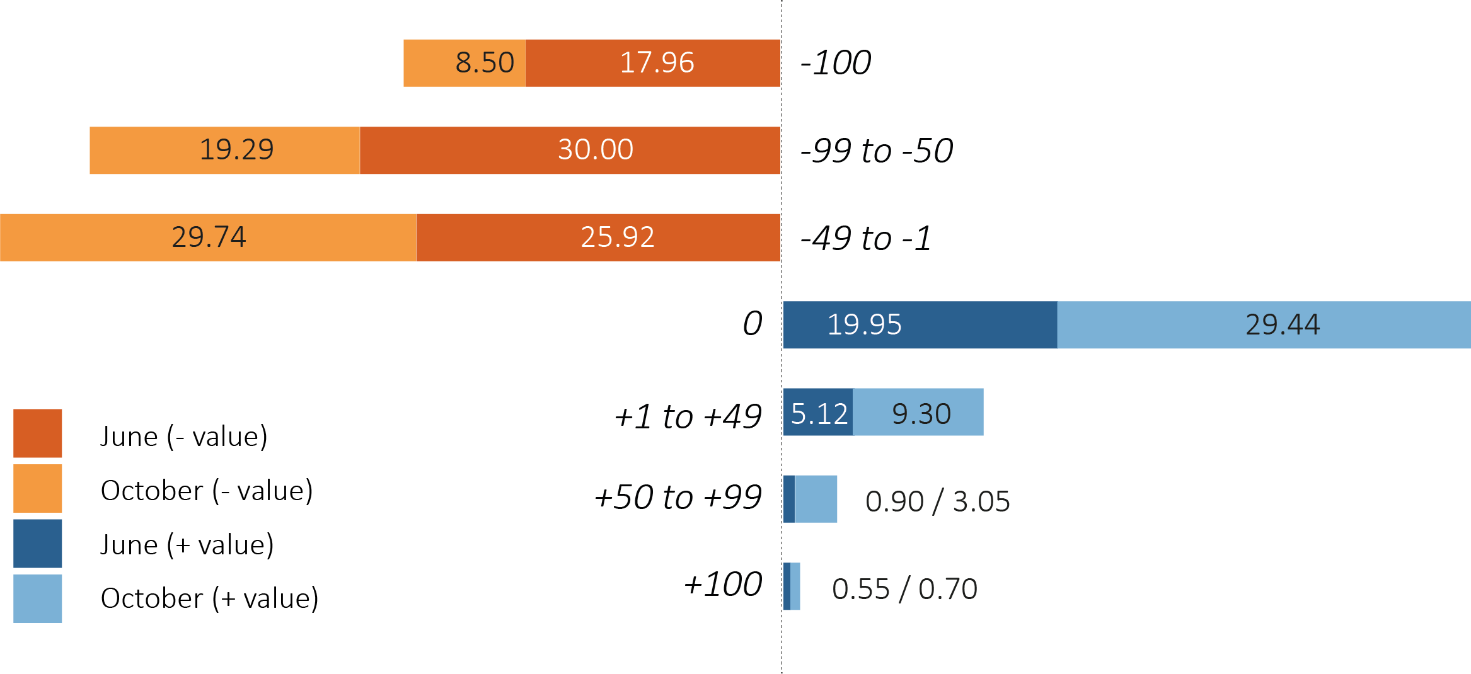


2.3. Average variation in conventional nuclear medicine diagnostic procedures by regional subgroup

|  | Region | Africa | Eastern Europe | Far East | Latin America | Middle East and South Asia | North America | South East Asia and the Pacific | Western Europe |
| --- | --- | --- | --- | --- | --- | --- | --- | --- | --- |
| **CNM June** | **-100** | -18.78 | -24.63 | -18.47 | -19.40 | -20.28 | -12.88 | -21.20 | -7.45 |
|  | **-99 to 50** | -29.95 | -26.12 | -17.12 | -38.61 | -27.61 | -38.64 | -33.15 | -21.96 |
|  | **-49/- 1** | -21.32 | -21.64 | -34.68 | -21.66 | -29.86 | -20.45 | -26.63 | -29.80 |
|  | **0** | -25.38 | -23.13 | -18.02 | -12.24 | -17.18 | -21.21 | -17.93 | -33.33 |
|  | **1 to 49** | 4.06 | 4.48 | 7.21 | 5.84 | 4.79 | 5.30 | 0.00 | 7.06 |
|  | **50 to 90** | 0.51 | 0.00 | 1.35 | 2.26 | 0.00 | 0.76 | 0.54 | 0.00 |
|  | **100** | 0.00 | 0.00 | 3.15 | 0.00 | 0.28 | 0.76 | 0.54 | 0.39 |
| **CNM October** | **-100** | -6.09 | -12.69 | -8.72 | -9.36 | -7.63 | -5.97 | -19.67 | -0.40 |
|  | **-99 to 50** | -12.69 | -44.78 | -14.68 | -23.03 | -21.47 | -27.61 | -7.10 | -8.10 |
|  | **-49 to 1** | -24.87 | -20.15 | -18.81 | -39.33 | -30.79 | -22.39 | -35.52 | -25.91 |
|  | **0** | -34.52 | 14.93 | 37.16 | 17.60 | 25.14 | 30.60 | 33.33 | 54.66 |
|  | **1 to 49** | 9.64 | 5.22 | 15.14 | 8.99 | 10.45 | 8.96 | 3.83 | 9.31 |
|  | **50 to 90** | 10.66 | 2.24 | 2.75 | 1.69 | 4.24 | 2.24 | 0.55 | 1.21 |
|  | **100** | 1.52 | 0.00 | 2.75 | 0.00 | 0.28 | 2.24 | 0.00 | 0.40 |

2.4. Average variation in conventional nuclear medicine diagnostic procedures by income subgroups.

2.5. Average variation in conventional nuclear medicine diagnostic procedures per exams

|  | | **Variations** | | | | | | | |
| --- | --- | --- | --- | --- | --- | --- | --- | --- | --- |
| **Exam** | **#** | **-100%** | **-99/ -50%** | **-49/ -1%** | **0%** | **+1/ +49%** | **+50/ +99%** | **+100%** | **Total <0** |
| **Bone scan** | | | | | | | | | |
| **June%** | **267** | 12.0 | 36.0 | 31.8 | 13.1 | 4.5 | 2.6 | 0.0 | 79.8 |
| **October%** | **263** | 5.7 | 21.3 | 34.6 | 22.8 | 9.5 | 5.7 | 0.4 | 61.6 |
| **Myocardial perfusion imaging** | | | | | | | | | |
| **June%** | **244** | 17.6 | 37.3 | 24.6 | 12.7 | 6.6 | 0.8 | 0.4 | 79.5 |
| **October%** | **244** | 8.6 | 20.9 | 34.8 | 19.3 | 11.5 | 4.5 | 0.4 | 64.3 |
| **Lung scans** | | | | | | | | | |
| **June%** | **213** | 23.0 | 28.6 | 19.3 | 23.5 | 4.7 | 0.5 | 0.5 | 70.9 |
| **October%** | **211** | 13.3 | 21.8 | 20.9 | 32.2 | 9.9 | 1.4 | 0.5 | 55.9 |
| **Renal scans** | | | | | | | | | |
| **June%** | **261** | 14.1 | 32.2 | 28.0 | 19.5 | 4.6 | 0.8 | 0.8 | 74.3 |
| **October%** | **260** | 6.2 | 19.2 | 35.0 | 27.7 | 8.9 | 2.3 | 0.8 | 60.4 |
| **Thyroid studies** | | | | | | | | | |
| **June%** | **261** | 16.9 | 30.3 | 27.6 | 18.8 | 5.4 | 0.8 | 0.4 | 74.7 |
| **October%** | **259** | 5.8 | 20.4 | 31.3 | 29.3 | 8.9 | 3.4 | 0.8 | 57.5 |
| **Sentinel node detection** | | | | | | | | | |
| **June%** | **194** | 18.6 | 22.7 | 28.3 | 23.2 | 6.2 | 0.5 | 0.5 | 69.6 |
| **October%** | **193** | 7.3 | 16.6 | 25.9 | 39.9 | 7.3 | 2.1 | 1.0 | 49.7 |
| **Parathyroid scan** | | | | | | | | | |
| **June%** | **241** | 20.3 | 25.7 | 24.5 | 23.2 | 4.6 | 0.4 | 1.2 | 70.5 |
| **October%** | **241** | 10.0 | 17.4 | 28.2 | 31.5 | 8.3 | 3.3 | 1.2 | 55.6 |
| **Brain studies** | | | | | | | | | |
| **June%** | **138** | 26.1 | 18.1 | 22.5 | 26.1 | 6.5 | 0.7 | 0.0 | 66.7 |
| **October%** | **137** | 16.1 | 12.4 | 21.9 | 36.5 | 11.0 | 1.5 | 0.7 | 50.3 |
| **Other studies** | | | | | | | | | |
| **June%** | **191** | 18.3 | 31.9 | 23.6 | 20.9 | 3.7 | 0.5 | 1.1 | 73.8 |
| **October%** | **193** | 7.8 | 20.2 | 28.5 | 32.6 | 8.8 | 1.6 | 0.5 | 56.5 |
| **Total Conventional Nuclear Medicine Studies** | | | | | | | | | |
| **June%** | **2010** | 18.0 | 30.0 | 25.9 | 19.6 | 5.1 | 0.9 | 0.5 | 73.9 |
| **October%** | **2001** | 8.5 | 19.3 | 29.7 | 29.4 | 9.3 | 3.0 | 0.7 | 57.5 |

1. **PET procedure**

3.1 Worldwide availability of PET scanners per income status

| Income groups | Number of PET scanners | PET scanners / million inhabitants | \| **% of total PET scanners** \| \| --- \| \|  \| |
| --- | --- | --- | --- | --- | --- |
| Low-income | 4 | 0.006 | 0.07 |
| Lower-middle-income | 452 | 0.155 | 7.97 |
| Upper-middle-income | 858 | 0.31 | 15.13 |
| High-income | 4,356 | 3.522 | 76.83 |

3.2. Average variation in PET procedures globally


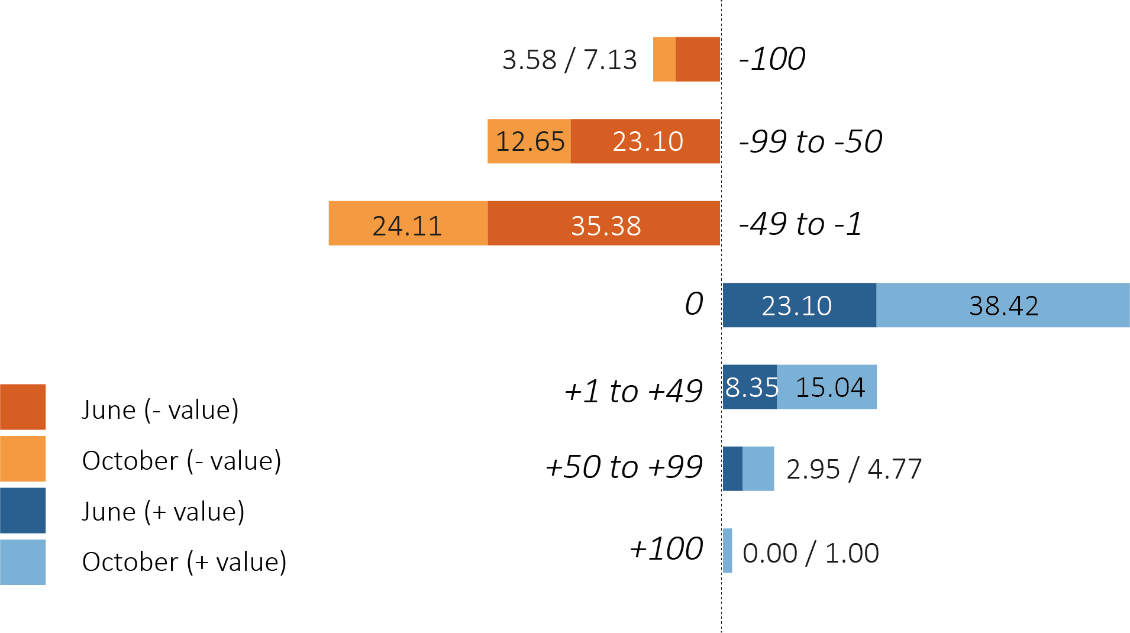


3.3. Average variation in PET procedures by regional subgroups

|  | Region | Africa | Eastern Europe | Far East | Latin America | Middle East and South Asia | North America | South East Asia and the Pacific | Western Europe |
| --- | --- | --- | --- | --- | --- | --- | --- | --- | --- |
| **PET June** | **-100** | 0.00 | -7.14 | -2.08 | -14.46 | -14.81 | 0.00 | -5.88 | 0.00 |
|  | **-99 to 50** | -18.42 | -17.86 | -20.83 | -39.76 | -19.75 | -29.41 | -35.29 | -7.69 |
|  | **-49/- 1** | -39.47 | -17.86 | -43.75 | -34.94 | -34.57 | -23.53 | -41.18 | -35.90 |
|  | **0** | 36.84 | 42.86 | 20.83 | 8.43 | 20.99 | 29.41 | 17.65 | 29.49 |
|  | **1 to 49** | 2.63 | 7.14 | 12.50 | 1.20 | 2.47 | 5.88 | 0.00 | 26.92 |
|  | **50 to 90** | 2.63 | 7.14 | 0.00 | 1.20 | 7.41 | 11.76 | 0.00 | 0.00 |
|  | **100** | 0.00 | 0.00 | 0.00 | 0.00 | 0.00 | 0.00 | 0.00 | 0.00 |
| **PET October** | **-100** | -2.63 | -8.00 | -2.08 | -5.43 | -7.23 | 0.00 | 0.00 | 0.00 |
|  | **-99 to 50** | -5.26 | -16.00 | -10.42 | -21.74 | -16.87 | -15.00 | -8.82 | -2.53 |
|  | **-49 to 1** | -36.84 | -32.00 | -33.33 | -26.09 | -18.07 | -25.00 | -29.41 | -11.39 |
|  | **0** | 36.84 | 40.00 | 37.50 | 31.52 | 27.71 | 45.00 | 41.18 | 55.70 |
|  | **1 to 49** | 10.53 | 0.00 | 14.58 | 6.52 | 20.48 | 5.00 | 20.59 | 26.58 |
|  | **50 to 90** | 5.26 | 4.00 | 2.08 | 7.61 | 4.82 | 10.00 | 0.00 | 3.80 |
|  | **100** | 2.63 | 0.00 | 0.00 | 1.09 | 4.82 | 0.00 | 0.00 | 0.00 |

3.4. Average variation in PET procedures by income subgroups

3.5. Average variation in PET procedures per exams

|  | | **Variations** | | | | | | | |
| --- | --- | --- | --- | --- | --- | --- | --- | --- | --- |
| **Exam** | **#** | **-100%** | **-99/ -50%** | **-49/ -1%** | **0%** | **+1/ +49%** | **+50/ +99%** | **+100%** | **Total <0** |
| **FDG (oncology)** | | | | | | | | | |
| **June%** | **161** | 3.1 | 21.1 | 44.1 | 17.4 | 9.9 | 4.4 | 0.0 | 68.3 |
| **October%** | **160** | 1.3 | 8.8 | 26.2 | 35.6 | 18.8 | 6.9 | 2.5 | 36.3 |
| **FDG (non-oncology)** | | | | | | | | | |
| **June%** | **115** | 10.4 | 27.0 | 28.7 | 26.1 | 5.2 | 2.6 | 0.0 | 66.1 |
| **October%** | **114** | 4.4 | 13.2 | 21.9 | 39.5 | 14.9 | 5.3 | 0.9 | 39.4 |
| **Gallium 68** | | | | | | | | | |
| **June%** | **85** | 7.1 | 25.9 | 29.4 | 28.2 | 7.1 | 2.4 | 0.0 | 62.4 |
| **October%** | **85** | 4.7 | 7.1 | 24.71 | 43.5 | 15.3 | 3.5 | 1.2 | 36.5 |
| **Other studies** | | | | | | | | | |
| **June%** | **46** | 13.0 | 15.2 | 32.6 | 26.1 | 13.0 | 0.0 | 0.0 | 60.9 |
| **October%** | **45** | 8.9 | 6.7 | 28.9 | 48.9 | 6.7 | 0.0 | 0.0 | 44.4 |
| **Total PET/CT studies** | | | | | | | | | |
| **June%** | **407** | 7.1 | 23.1 | 35.4 | 23.1 | 8.4 | 2.9 | 0.0 | 65.6 |
| **October%** | **409** | 3.6 | 12.6 | 24.1 | 38.4 | 15.0 | 4.8 | 1.4 | 40.3 |

1. Radionuclide therapies

4.1. Average variation in therapeutic procedures globally


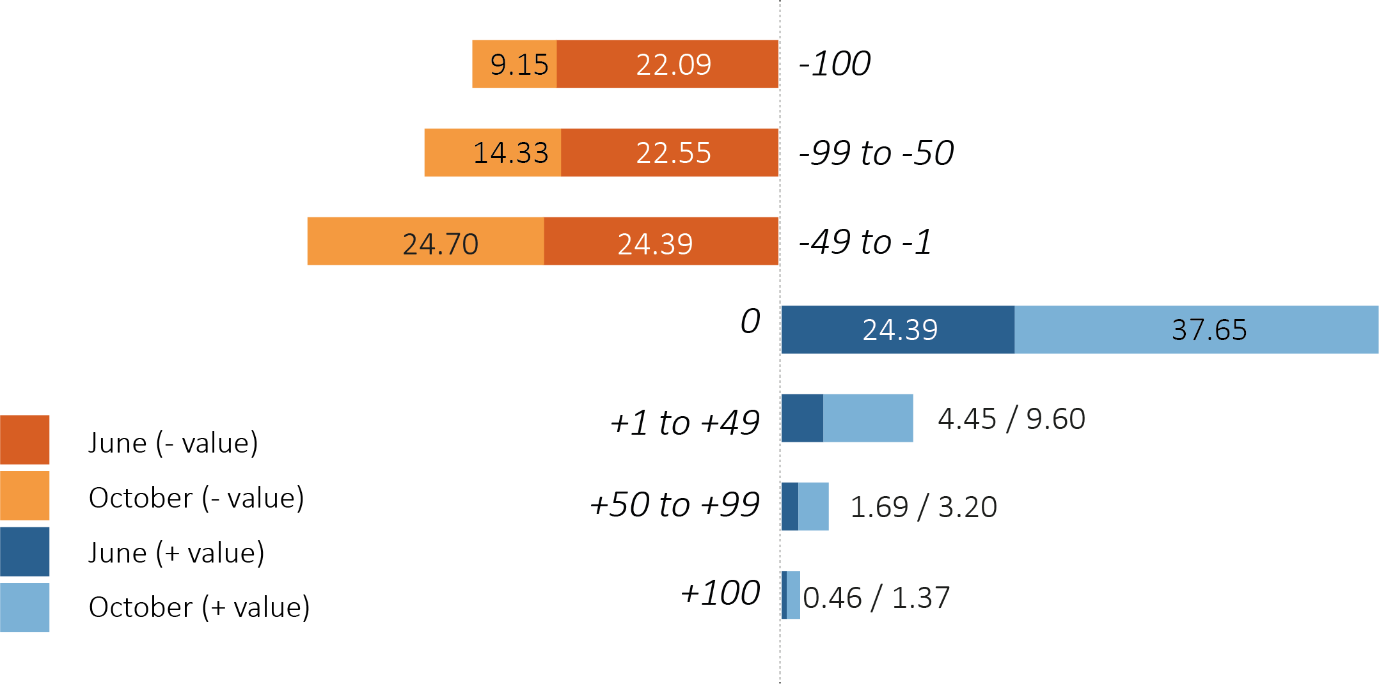


4.2. Average variation in therapeutic procedures by regional subgroups

|  | Region | Africa | Eastern Europe | Far East | Latin America | Middle East and South Asia | North America | South East Asia and the Pacific | Western Europe |
| --- | --- | --- | --- | --- | --- | --- | --- | --- | --- |
| **RT June** | **-100** | -28.41 | -20.00 | -19.35 | -28.78 | -22.45 | -21.05 | -24.32 | -7.77 |
|  | **-99 to 50** | -17.05 | -15.00 | -25.81 | -22.30 | -26.53 | -15.79 | -37.84 | -11.65 |
|  | **-49/- 1** | -20.45 | -30.00 | -24.19 | -22.30 | -27.89 | -31.58 | -21.62 | -25.24 |
|  | **0** | 25.00 | 30.00 | 14.52 | 20.14 | 21.77 | 31.58 | 16.22 | 42.72 |
|  | **1 to 49** | 2.27 | 0.00 | 8.06 | 6.47 | 0.68 | 0.00 | 0.00 | 11.65 |
|  | **50 to 90** | 4.55 | 5.00 | 8.06 | 0.00 | 0.68 | 0.00 | 0.00 | 0.00 |
|  | **100** | 2.27 | 0.00 | 0.00 | 0.00 | 0.00 | 0.00 | 0.00 | 0.97 |
| **RT October** | **-100** | -10.23 | -5.00 | -4.84 | -15.60 | -6.12 | -19.05 | -14.86 | -0.97 |
|  | **-99 to 50** | -11.36 | -20.00 | -20.97 | -14.89 | -17.69 | -14.29 | -16.22 | -4.85 |
|  | **-49 to 1** | -22.73 | -40.00 | -19.35 | -26.95 | -27.89 | -28.57 | -31.08 | -13.59 |
|  | **0** | 28.41 | 35.00 | 30.65 | 29.79 | 36.05 | 38.10 | 36.49 | 64.08 |
|  | **1 to 49** | 14.77 | 0.00 | 14.52 | 9.93 | 6.80 | 0.00 | 1.35 | 15.53 |
|  | **50 to 90** | 7.95 | 0.00 | 6.45 | 1.42 | 4.76 | 0.00 | 0.00 | 0.97 |
|  | **100** | 4.55 | 0.00 | 3.23 | 1.42 | 0.68 | 0.00 | 0.00 | 0.00 |

4.3. Average variation in therapeutic procedures by income subgroups

4.3. Average variation per radionuclide therapies

|  | | **Variations** | | | | | | | |
| --- | --- | --- | --- | --- | --- | --- | --- | --- | --- |
| **Treatment** | **#** | **-100%** | **-99/ -50%** | **-49/ -1%** | **0%** | **+1/ +49%** | **+50/ +99%** | **+100%** | **Total <0** |
| **Thyroid cancer** | | | | | | | | | |
| **June%** | **180** | 21.7 | 25.6 | 25.6 | 19.4 | 5.0 | 2.2 | 0.6 | 72.8 |
| **October%** | **183** | 7.1 | 14.8 | 26.2 | 33.3 | 11.5 | 5.4 | 1.6 | 48.1 |
| **Thyroid benign** | | | | | | | | | |
| **June%** | **197** | 25.9 | 26.4 | 24.9 | 16.8 | 3.6 | 2.0 | 0.5 | 77.1 |
| **October%** | **198** | 8.1 | 18.7 | 27.8 | 30.3 | 9.1 | 3.5 | 2.5 | 54.6 |
| **PRRT** | | | | | | | | | |
| **June%** | **66** | 12.1 | 15.2 | 33.3 | 30.3 | 4.6 | 3.0 | 1.5 | 60.6 |
| **October%** | **66** | 6.1 | 3.0 | 25.8 | 51.5 | 10.6 | 1.5 | 1.5 | 34.9 |
| **PSMA** | | | | | | | | | |
| **June%** | **53** | 20.8 | 15.1 | 20.8 | 32.1 | 9.4 | 1.9 | 0.0 | 56.6 |
| **October%** | **53** | 11.3 | 7.6 | 18.9 | 49.1 | 11.3 | 1.9 | 0.0 | 37.7 |
| **Bone pain palliation** | | | | | | | | | |
| **June%** | **66** | 15.2 | 27.3 | 22.7 | 30.3 | 4.6 | 0.0 | 0.0 | 65.2 |
| **October%** | **66** | 12.1 | 15.2 | 22.7 | 47.0 | 3.0 | 0.0 | 0.0 | 50.0 |
| **SIRT** | | | | | | | | | |
| **June%** | **41** | 17.1 | 14.6 | 19.5 | 46.3 | 2.4 | 0.0 | 0.0 | 51.2 |
| **October%** | **41** | 9.8 | 14.6 | 9.8 | 53.7 | 9.8 | 2.4 | 0.0 | 34.2 |
| **Radiosynovectomy** | | | | | | | | | |
| **June%** | **23** | 43.5 | 8.7 | 17.4 | 30.4 | 0.0 | 0.0 | 0.0 | 69.6 |
| **October%** | **23** | 21.7 | 21.7 | 21.7 | 26.1 | 8.7 | 0.0 | 0.0 | 65.2 |
| **Other treatments** | | | | | | | | | |
| **June%** | **26** | 30.8 | 19.2 | 15.4 | 30.8 | 3.9 | 0.0 | 0.0 | 65.4 |
| **October%** | **26** | 15.4 | 11.5 | 30.8 | 26.9 | 11.6 | 3.9 | 0.0 | 57.7 |
| **Total treatments** | | | | | | | | | |
| **June%** | **652** | 22.1 | 22.5 | 24.4 | 24.4 | 4.4 | 1.7 | 0.5 | 69.0 |
| **October%** | **656** | 9.1 | 14.3 | 24.7 | 37.7 | 9.6 | 3.2 | 1.4 | 48.2 |

1. **Supply**

Percentage of the responses indicating sufficient (yes) or insufficient (no) supply of materials in June and in October 2020 (not applicable answers are not indicated).


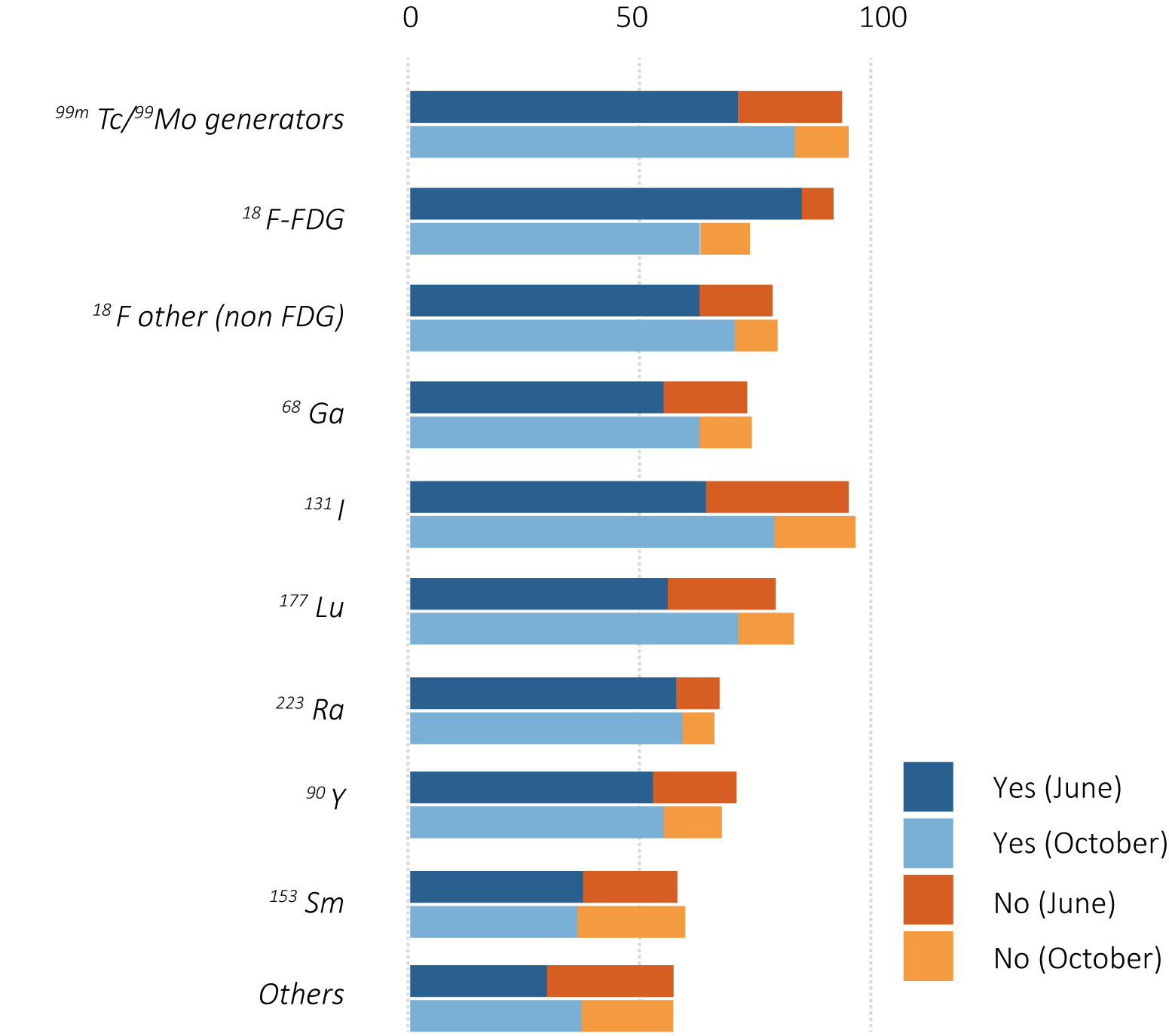

Supplement: Supplementary file 1 — Supplementary file1 (DOCX 292 KB) [file 259_2021_5444_MOESM1_ESM.docx]
