# Supplementary material for: Changes in the global impact of COVID-19 on nuclear medicine departments during 2020: an international follow-up survey
Source: Eur J Nucl Med Mol Imaging. 2021 Jun 19;48(13):4318–30. doi: 10.1007/s00259-021-05444-7 (PMC8214460; doi:10.1007/s00259-021-05444-7)
Supplement: Supplementary file 2 — Supplementary file2 (DOCX 40.0 KB) [file 259_2021_5444_MOESM2_ESM.docx]

**NMDI-COVID**

**One Year Later: Global Impact of COVID-19 on Nuclear Medicine Departments; an International Follow-up Survey**

**Appendix: Table of Contents**

NMDI-COVID Investigators Group Pages 2-9

Country Participation Page 10

**NMDI-COVID Investigators Group**

**Steering Committee:** Diana Paez (IAEA section head); (alphabetically) Partha Choudhary Ken Herrmann, Noura El-Haj, Enrique Estrada, Lutz S. Freudenberg, Lynne Gatward, Francesco Giammarile, Greta Ladaa, Miriam Mikhail, Maria Camila Navarro, Pilar Orellana, Olivier Pellet, Elena Sanchez-Romero

**Information Technology and Statistics Committee:** Yaroslav Pynda (chair); (alphabetically): Gerd Hinterleitner, Olga Morozova

**Members (alphabetically by country and last name)**

**Algeria:** Salah Bouyoucef, Abdelkader Medjahedi

**Argentina:** Mariela Agolti, Roberto Nicolas Aguero, Raul Cabrejas, Veronica Cabrejas, Carlos Castro, Gonzalo Costa, Adriana Elizondo, Fernando Faccio, Adolfo Facello, Horacio Glait, Carolina Keimichel, Soledad Machello, Daniel Nespral, Patricia Parma, Marianela Rotania, Daniel B Schere, Sonia Traverso

**Australia:** Dale Bailey, Lauren Marks, Andrew Scott, Kim Taubman

**Austria:** Rainer LIPP, Siroos Mirzaei

**Belgium:** Olivier Gheysens

**Bolivia:** Rosario Urquieta

**Bosnia - Herzegovina:** Belkisa Izic, Renata Milardovic, Fatima Mujaric

**Brazil:** Adelanir Barroso, Mateos Bogoni, Juliano Cerci, Jader Cunha de Azevedo, Uysha de Souza Fonda, Elba Etchebehere, Marycel Fiigols de Barboza, Andréia Fischer, Rafael Lopes, Margaret Masukawa, Sara Melo Macedo Santana, Sonia Moriguchi, Andre Oliveira, Lima Ronaldo, Paulo Henrique Rosado de Castro, Marcelo Silva, Marcia Tavares

**Bulgaria:** Marina Dyankova, Irena Kostadinova, Gabriela Mateva

**Cambodia:** Sarameth Thou, Soley Varoeun

**Canada:** Rick Dubeau, Rob Beanlands

**Chile:** Carmen Concha, Cesar Lovera, Teresa Massardo, Pilar Orellana, Byron Riedel, Luis Ignacio Salazar Vargas, Patricio Weitz

**Colombia:** Marylin Acuña Hernández, Sonia Merlano-gaitan, Tatiana Morales, Ramon Murgueitio, Osacar Alejandro Osorio Echeverry, Alvaro Pacheco, Jairo Poveda, Juan Carlos Rojas M, Ivan Salamanca

**Costa Rica:** Mauricio Acuña, Gabriel Castro, Erasmo Antonio Serrano

**Croatia:** Maja Franceschi

**Cuba:** Yamilé Peña, Alejandro Perera Pintado

**Cyprus:** Savvas Frangos

**Czech Republic:** Otto Lang

**Dominican Republic:** Rosangie Del Castillo

**Ecuador:** María Augusta Charvet Araque, Andrea Martinez, Mario Pérez, Carlos Regalado, Erick Rundo, Gabriela Segura

**Egypt:** Aya Abaza, Elshaymaa Hussein, Reda Hammad

**El Salvador:** Claudia Figueroa

**Ethiopia:** Rahel Leta, Solomon Welelu

**Fiji:** Camari Waqanisau

**Finland:** Hanna Mussalo

**France:** Nicolas Aide, Olivier Lairez

**Germany:** Ken Herrmann, Klemens Scheidhauer, Roman P. Schneider

**Ghana:** Alfred Ankrah

**Greece:** Vasiliki Chatzipavlidou, Panagiotis Georgoulias

**Hong Kong SAR (China):** Chiu Ming Lok

**Hungary:** Ildikó Garai, Zoltan Toth

**India:** Partha Choudhury, Manoranjan Chowhan, Vankadari Kpusik, Parul Mohan, Tapan Patel, Neelendra Pathak, Sudatta Ray, Sunita Sonavane, Madhur Lumar Srivastava, Sudhanshu Tonpe

**Indonesia:** Hendra Budiawan, Ayu Rosemeilia Dewi, Edison Edison, Aisyah Elliyanti, Andika Hananto Gunawan, Nur Hidayati, Febby Hutomo, Yustia Tuti Jelani, Yulia Kurniawati, Ryna Martiana, Moh Shofi Nur Utami, Djoko NK Prawiro, Pingkan Siregar, Erwin Affandi Soeriadi, Endah Indriani Soeriadi, Indriani Wahyono, Ryan Yudistiro

**Iraq:** Amjed Albadr, Muntather Habeeb Albo Saabar, Rafid Al-Tuma

**Islamic Republic of Iran:** Atena Aghaee, Mahmood Mohammadi Sadr, Seyed Rasoul Zakavi

**Israel:** Zvi Bar-Sever, Simona Ben-Haim, Sigalit Haruz-Wachitz, Victoria Koulikov, Mordechai Lorberboym

**Italy:** Chiara Maria Grana, Critina Nanni, Carmela Nappi, Alberto Signore, Luca Tagliabue, Annibale Versari

**Japan:** Koichiro Kaneko, Masami Kawamoto, Kumiko Kono, Nobuyuki Kosaka, Ichiei Kuji, Ryogo Minamimoto, Tsutomu Miyauchi, Hiroshi Toyama

**Jordan:** Shadia Abu Baker, Mais Al Halaseh, Abdullah Al Zreiqat, Khaled Alkhawaldeh, Qabas Alreuwaishedi, Shahed Obeidat

**Kenya:** Khalid Makhdomi, David Wanjeh

**Kuwait:** Hanan Aldousari, Sarah Murad, Wafaa Yehia

**Latvia:** Marika Kalnina

**Lebanon:** Arpyk Adourian, Mahmoud El Najami

**Libya:** Ahmed Y.M. Ben Aouun

**Lithuania:** Donatas Vajauskas

**Malaysia:** Zool Hilmi Awang, Farahnaz Binti Muhamed Aslum Khan, Sunil Chopra, Abdullah Daud, Hazlin Hashim, Phay Phay Khor, Mohamad Aminudin Said, Teik-Hin Tan

**Mauritania:** Mohamed Lemine Dieng

**Mauritius:** Mohammad Aaftaab Allarakha, Amreeta Mangatha, Harrydeo Sonea

**Mexico:** Estrella Aguilera Hernández, Angelica Esperanza Arellano Zarate, Estrella Avila Ramirez, Erika Fernanda Barragan Pineda, Jose Angel Barrera Romero, Uvi Cancino Ramos, Cicilia Carreras, Gisela Estrada, Manlio Gerardo Gama-Moreno, Arturo Garcia, Virginia Garcia Quinto, José Ruben Gómez Garibo, Jorge Gerardo Guevara Villamar, Sevastian Medina, Roberto Mendiola, Monica Janet Mendoza Figueroa, Rafael Humberto Morales Murguía, Eurídice Rioja Guerrero, Belen Rivera Bravo, Edgar Omar Rodriguez Rojas, Nicolas Sanchez, Sigelinda Sandoval Borrego, Jose Antonio Serna, Luis Vargas, Katya Vazquez, Jose Luis Velasquez-Fajardo, Amanda Catalina Villalobos Cedano, Mateos

**Mongolia:** Enkhtuya Byambajav

**Montenegro:** Ljiljana Bojic

**Morocco:** Nouzha Ben-Rais

**Myanmar:** Thinn Thinn Myint, May Wathan Myo, Su Thet Oo, Win Naing Phyo, Su Yin Phyo Shein, Soe Myat Win, Htin Zar

**Nepal:** Anu Bhattarai, Sujita Rajbanshi

**Netherlands:** Andor Glaudemans, Reiny Kooistra, Riemer Slart

**Nicaragua:** Brisa Álvarez, Teresa Isabel Cuadra Diaz

**Niger:** Idrissa Adamou Soli

**Nigeria:** Musa Abdulhamid, Akintunde Orunmuyi

**Oman:** Anjali Jain, Zabah Jawa, Naima Tag

**Pakistan:** Akhtar Ahmed, Ayesha Ammar, Kahkashan Bashir Mir, Shazia Fatima, Babar Khan, Fateh Muhammad, Muhammad Atif, Nayyar Rubab, Shabana Saeed, Ayesha Syeda, Hira Tahir, Farzeen Zehra, Sabeen Zia

**Palau**: Ngirachisau Mekoll

**Panama:** Yariela Edith Herrera Malo

**Paraguay:** Graciela Giménez Ortigoza, Carlos Laterza, Maria Gloria Pedrozo Arrua, Luciano Recalde

**People's Republic of Bangladesh:** Rawnak Afrin, Afroza Akhter, Faria Nasreen, Azmal Kabir Sarker, Shaila Sharmin, Nasreen Sultana

**People's Republic of China:** Xinming Zhao

**Peru:** Arlene Garcia, Danfer Huapaya, Rosanna Morales, Evert José Reyes Armas, Patricia Saavedra

**Philippines:** Emerita Barrenechea, Asela Barroso, Patricia Bautista-Peñalosa, Maria Teresa Borras, Susan Camomot, Kristine Anne Marie Carlos, Vernie Convicto, Chris Eduarte, Melvin Estonactoc, Ken G, Rudolf Kuhn, Maria Lourdes Lacanilao, Aida Lobriguito, Jerry Obaldo, Ruben Ogbac, Michele Ogbac, Eduardo Erasto Ongkeko, Rizalina Osorio, Petronilo Parungao, Arnel Pauco, Jose Eduardo Rondain, Carlo Jose San Juan, Jonas Santiago, Marie Rhiamar Sauler-Gómez, Charles Tan, Marcelino Tanquilut

**Poland:** Jolanta Kunikowska

**Portugal:** Ana Isabel Santos

**Puerto Rico (United States):** Angelys Monell

**Qatar:** Rana Al-Araer

**Republic of Korea:** Henry Bom, Keon Wook Kang

**Republic of Kyrgyzstan:** Aliya Kadyrova, Begaiym Kulbaeva

**Republic of Moldova:** Olga Simionica

**Republic of North Macedonia:** Ana Ugrinska

**Republic of Slovenia:** Ivana Žagar

**Romania:** Iulia Andreea Chiriac, Mihaela-Georgiana Ilie-Lepus, Raluca Mititelu, Ana Maria Statescu

**Russian Federation:** Elena Slobina, Viacheslav Sukhov

**Saudi Arabia:** Muaadh Alasbahi, Abdelbaset Alrawashdeh, Mousa Bakkari, Walaa Hussein Eltahir Elzuber

**Senegal:** Rosalie Sara Senghor

**Serbia:** Milan Bozinovic, Dragana Sobic Saranovic

**Singapore:** Laurentcia Arlany, Anbalagan Kannivelu, David Ng, Somanesan S

**Slovak Republic:** Andrej Vondrak

**South Africa:** George Bennie, Anine de Wet, Jo-Anne Dreyer, Werner du Toit, Osayande Evbuomwan , Bawinile Hadebe, Leshae Jaiswar, Phumelele Khanyile, Janke Kleynhans, Adele Koegelenberg, Nisaar Korowlay, Greta Lange, Andre Lourens, Lizette Louw, Katarina Milos, Moshe Modiselle, Stuart More, Mike Sathekge, Samuel Teye, Hilton Thomas, Annalize Van Staden, Noluthando Zondi

**Spain:** Ignasi Carrio, Jose Garcia, Amelia Jimenez-Heffernan, Antonio Maldonado, Edel Noriega Álvarez, Iván Pañuelas, Miguel Sánchez Torrente

**Sri Lanka:** Damayanthi Nanayakkara, Aruna Pallewatte

**Sweden:** Anders Sundin

**Switzerland:** Gerhard Goerres, John Prior

**Taiwan (China):** Chao-Jung Chen, Chuang-Hsin Chiu, Shihchin Chou, Guang-Uei Hung, Yueh Lee, Neng-chuan Tseng, Ruoh-Fang Yen

**Tanzania:** Tausi Maftah. Faraji Sabaya

**Togo:** Aliou Bagnon

**Tunisia:** Aida Mhiri

**Turkey:** Zerrin Dede, Kemal Metin Kir

**Uganda:** Zerida Muyinda

**Ukraine:** Natalia Arkhanhelska, Pavlo Korol, Yuliia Yakymiv

**United Arab Emirates:** Suad Abdulrasool, Raqwana Baharoon

**United Kingdom:** Jamshed Bomanji, Emily Fittock, Maria Morais, Salma Subhani, Sobhan Vinjamuri

**United States of America:** Penelope Agodoa, John Austen, Danny Basso, Michael Bandina, Adam Brown, Jeremie Calais, Angela Callahan, Richelle Campbell, Shaun Caraway, Patricia Clements, Raquel Custodio, David D, Robert Gibbs, Harkanwar Gill, Peter Giuliano, Abbie Grancorvitz, Armando Guerrero, Stephanie Hamer, Karen Hassen, Sara Hauser, Beth Jines, Valerie Konon, Lisa Leach, Tony M, Jonathan McConathy, Melisa Mendez, Elia Meza, Dori Nelson, Alyssa O’Donnell, Johnny Ortega, Anne Parker, Neil Petry, Tonee Porter, Kirwan Price, Nancy Quachang, Jennifer Scheler, Tim Schultz, Paul Searfoss, Kimberly Shelton, Shyam Srinivas, Lucy Tahara, Eric Teal, Louise Thomson, Donald Trepashko, Dave Woods, Adrienne

**Uruguay:** Mario Beretta, Rodolfo Ferrando, Luisa Portugal, Susana Seoane

**Yemen:** Saif Ahmed Ali Abdulhadi, Abdo Al-Qubati, Esam Ahmed Ali Al-Shami, Aiman Zain

**Zambia:** Aubrey Musonda

**Zimbabwe:** Bonani Audrey Tuturu

**Methods for Data Collection, Site Entry, and Data Analysis**

We used IAEA-specific coding for world regions. Specifically, region participation included the following countries:

- **Africa** (n=19 countries): Algeria, Egypt, Ethiopia, Ghana, Kenya, Libya, Mauritania, Mauritius, Morocco, Niger, Nigeria, Senegal, South Africa, Tanzania, Togo, Tunisia, Uganda, Zambia, Zimbabwe
- **Eastern Europe** (n=18 countries): Bosnia – Herzegovina, Bulgaria, Croatia, Czech Republic, Estonia, Hungary, Latvia, Lithuania, Montenegro, Poland, Republic of Kyrgyzstan, Republic of Moldova, Republic of North Macedonia, Republic of Slovenia, Romania, Russian Federation, Serbia, Ukraine
- **Far East** (n=7 countries): Cambodia, Japan, Mongolia, People's Republic of China (Including Hong Kong SAR and Taiwan), Philippines, Republic of Korea, Vietnam
- **Latin America** (n=16 countries): Argentina, Bolivia, Brazil, Chile, Colombia, Costa Rica, Cuba, Dominican Republic, Ecuador, El Salvador, Mexico, Nicaragua, Panama, Paraguay, Peru, Uruguay
- **Middle East and South Asia** (n=16): India, Iraq, Islamic Republic of Iran, Israel, Jordan, Kuwait, Lebanon, Nepal, Oman, Pakistan, People's Republic of Bangladesh, Qatar, Saudi Arabia, Sri Lanka, United Arab Emirates, Yemen
- **North America** (n=2 countries): Canada, United States of America (including Puerto Rico)
- **South East Asia and the Pacific** (n=8 countries): Australia, Fiji, Indonesia, Malaysia, Myanmar, Palau, Singapore, Thailand
- **Western Europe** (n=15 countries): Austria, Belgium, Cyprus, Finland, France, Germany, Greece, Italy, Netherlands, Portugal, Spain, Sweden, Switzerland, Turkey, United Kingdom
