# Supplementary material for: Changes in the global impact of COVID-19 on nuclear medicine departments during 2020: an international follow-up survey
Source: Eur J Nucl Med Mol Imaging. 2021 Jun 19;48(13):4318–30. doi: 10.1007/s00259-021-05444-7 (PMC8214460; doi:10.1007/s00259-021-05444-7)
Supplement: Supplementary file 3 — Supplementary file3 (PDF 428 KB) [file 259_2021_5444_MOESM3_ESM.pdf]

Impact of COVID-19 on Nuclear Medicine Practices Worldwide

The COVID-19 pandemic has led to profound disruptions in the delivery of healthcare and nuclear medicine is no exception.

In March 2020 the IAEA conducted a survey to evaluate the impact of the pandemic on nuclear medicine departments, published in JNM in September 2020 (PMID: 32709733).

A follow-up survey is now being implemented to understand and identify the long-term effects of this pandemic, evaluate them and propose global solutions.

The higher the participation the more meaningful the results will be to support countries to identify challenges and address them. We kindly ask you for 5 minutes of your time to fill out this survey.

Organization

* Country/Administrative Area — Choose one of the following...

Province/State/Territory

* City

Name of Institution

* Type of institution
- ☐ Hospital
- ☐ University hospital
- ☐ Private practice

About Yourself

Your First/Given Name

Your Family Name/Surname

If you wish to stay in touch with us then please share your email here

Your profession (please choose one main one which reflects your major specialty)?

- Nuclear medicine specialist
- Radiologist
- Medical Physicist
- Radiopharmacist
- Technologist
- Other

Other

Nuclear Medicine Practice

What was the percentage of outpatients in your department before COVID-19?

Was there a change in the percentage of outpatients in your department in June 2020 compared to January 2020 or prior to the COVID pandemic?

- Increased
- Decreased
- No change

Was there a change in the percentage of outpatients in your department in October 2020 compared to January 2020 or prior to the COVID pandemic?

- Increased
- Decreased
- No change

Do you perform general nuclear medicine (SPECT) procedures in your department?

- Yes
- No

Do you perform PET scans in your department? ◯ Yes ◯ No

Do you perform radionuclide therapies in your department? ◯ Yes ◯ No

Compared to usual activity (January 2020 – prior to the begin of the pandemic) : How did SPECT procedures change?

-100% (complete absence of exams after COVID),
0% (exams as usual)
+100% (double the number of exams)
Not applicable if these studies are not performed in your department

	Yes	June 2020	Oct. 2020
Bone scans	☐		
Myocardial perfusion imaging	☐		
Lung scans	☐		
Renal scans	☐		
Thyroid studies	☐		
Sentinel node detection	☐		
Parathyroid scan	☐		
Brain studies	☐		
Other (e.g. MUGA, salivary, GE reflux, gastric emping etc)	☐		

emping etc)

Compared to usual activity (January 2020 – prior to the begin of the pandemic) : How did PET procedures change?

-100% (complete absence of exams after COVID),
0% (exams as usual)
+100% (double the number of exams)
Not applicable if these studies are not performed in your department

	Yes	June 2020	Oct. 2020
PET/CT FDG (oncology)	☐		
PET/CT FDG (non-oncology)	☐		
PET/CT Gallium 68	☐		
Others (e.g. C11 based, N13, O15, F choline, etc)	☐		

Compared to usual activity (January 2020 – prior to the begin of the pandemic) : How did radionuclide therapy procedures change?

-100% (complete absence of exams after COVID),
0% (exams as usual)
+100% (double the number of exams)
Not applicable if these studies are not performed in your department

	Yes	June 2020	Oct. 2020
Thyroid (malignant)	☐		
Thyroid (benign)	☐		

Peptide receptor radionuclide therapy (PRRT)	☐		
Prostate-specific membrane antigen (PSMA)	☐		
Bone pain palliation	☐		
Selective internal radiation therapy (SIRT)	☐		
Radiosynoviorthesis (RSO)	☐		
Other	☐		

Personal Protective Equipment (PPE)

Was there a shortage of PPE in your department for you and your employees in June 2020?

◯ Yes ◯ No

Was there a shortage of PPE in your department for you and your employees in October 2020?

◯ Yes ◯ No

For how many days do you currently have personal protective equipment on stock for you and your employees?

◯ Less than 2 days
◯ 2-7 days
◯ 7-14 days
◯ 14-28 days
◯ more than 28 days

Employee Health and Organizational Adjustments to the COVID-19 Crisis

Have employees in your department been infected with COVID-19?

◯ No

[illegible]

- ◯ Yes, less than 20% of the employees
- ◯ Yes, 20%-40% of the employees
- ◯ Yes, 40%-60% of the employees

Have employees in your department been relocated to other clinical departments or areas?

- ◯ No
- ◯ Yes, less than 20% of the employees
- ◯ Yes, 20%-40% of the employees
- ◯ Yes, 40%-60% of the employees

If the answers to the previous question is yes; Have they returned to your department?

- ◯ No
- ◯ Yes, less than 20% of the employees
- ◯ Yes, 20%-40% of the employees
- ◯ Yes, 40%-60% of the employees

Were there changes in the typical working hours?

- ◯ No
- ◯ Yes, less than 20% of the employees
- ◯ Yes, 20%-70% of the employees
- ◯ Yes, more than 70% of the employees

Have there been lay-offs or ford lodge in your department due to COVID-19?

- ◯ No
- ◯ Yes, less than 20% of the employees
- ◯ Yes, 20%-70% of the employees
- ◯ Yes, more than 70% of the employees

Are there any other organizational adjustments to the COVID-19 crisis in your department?

Supply of radioactive and other essential materials

Did you adjust your orders of Mo/Tc generators?

- ◯ No
- ◯ Yes, we have unsubscribed our generators
- ◯ Yes, we are still ordering less than 20% of our normally ordered activity

◯ Yes, we are still ordering 20-70% of our normally ordered activity

◯ Yes, we are still ordering >70% of our normally ordered activity

Has your department's supply of the following essential materials been sufficient?
Not applicable if these studies are not performed in your department

	Yes	June 2020	Oct. 2020
Mo/Tc generators	☐		
18 F-FDG	☐		
Other 18 F labelled tracers	☐		
Gallium 68 Generators	☐		
Iodine 131	☐		
Lutetium 177	☐		
Radium 223	☐		
Yttrium 90	☐		
Samarium 153	☐		
Cold kits	☐		
Others (e,g. P32, Er169, Re186 ...)	☐		

Anticipations and opportunities

For how long do you anticipate the COVID-19 pandemic will affect your department in relation to SPECT studies?

- ◯ 6 months
- ◯ 1 year
- ◯ 5 years
- ◯ more than 5 years
- ◯ we will not be recovered

For how long do you anticipate the COVID-19 pandemic will affect your department in relation to PET/CT studies?

- ◯ 6 months
- ◯ 1 year
- ◯ 5 years
- ◯ more than 5 years
- ◯ we will not be recovered

What are your fears concerning the current situation?

- ☐ Not being able to pay debts
- ☐ Decrease in salaries
- ☐ Reduction of staff
- ☐ Not being able to recover the usual number of procedures
- ☐ Not being able to get the needed supplies
- ☐ Other please specify

Please specify other fears __________

What opportunities do you see in the current situation?

- ☐ Increases in the use of technology for virtual meetings
- ☐ Optimizing use of resources
- ☐ Reduction of staff
- ☐ Increase in the use of PET technology
- ☐ Resilience in crisis management
- ☐ Other opportunities

Please specify other opportunities __________
